# Surgical Residents' Feedback Perceptions: A Scoping Review on Gaps and Improvements

**DOI:** 10.1111/tct.70323

**Published:** 2025-12-15

**Authors:** Carlos Dario da Silva Costa, Gabriela Gouvea Silva, Emerson Roberto dos Santos, Alba Regina de Abreu Lima, Vânia Maria Sabadoto Brienze, Denise Cristina Móz Vaz Oliani, Antonio Hélio Oliani, Júlio César André

**Affiliations:** ^1^ Center for Studies and Development of Health Education—Faculty of Medicine of São José do Rio Preto (CEDES/FAMERP) São José do Rio Preto Brazil; ^2^ Faculty of Medicine of São José do Rio Preto (FAMERP), Brazil, and University Hospital Center Cova da Beira University of Beira Interior Covilhã Portugal

**Keywords:** feedback, internship and residency, medical education, perception, surgery

## Abstract

**Background:**

Feedback is a pivotal instrument for learning and performance enhancement in medical and surgical training. Its established importance for resident development, aiming for highly qualified patient care, faces delivery and utilisation challenges, shifting focus to active learner reception. A critical gap exists in understanding surgical residents' perceptions of this essential process.

**Objective:**

This study aims to systematically identify and map available data regarding surgical residents' feedback perceptions during training, thoroughly analysing findings and delineating existing knowledge gaps.

**Methods:**

Following the Arksey and O'Malley and JBI methodology, a comprehensive search across Medline, Directory of Open Access Journals (DOAJ), Directory of Open Access Scholarly Resources (ROAD), Academic Search Premier (ASP), BioMed Central Open Access (BMC) and Wiley–Blackwell (Wiley) included studies on surgical residents' feedback perceptions (attitudes, experiences, values, beliefs, satisfaction and reported impact). Data charting involved both quantitative and qualitative analyses.

**Results:**

Twelve articles (2017–2024), predominantly United States‐based, were included. Residents consistently valued feedback for development and confidence, preferring immediate, verbal and face‐to‐face delivery, ideally during or directly following a procedure. Common concerns included low frequency, lack of specificity or explicit labelling and delayed provision leading to perceived irrelevance. Influential factors encompassed timing, the learning environment, source credibility (senior residents often preferred) and preceptor personal traits. Critically, the direct impact on learning progress and skill development was often underexamined.

**Conclusion:**

The current evidence based on surgical residents' feedback perceptions is limited by methodological heterogeneity, reliance on retrospective designs and insufficient direct measurement of its actual impact. Resident–preceptor perception discrepancies persist, alongside inadequate detail on feedback characteristics. Thus, standardised, comprehensive and impact‐focused research is critically needed to enhance surgical training feedback practices, ultimately contributing to improved patient care.

**Trial Registration:** Not applicable

**Abbreviations**
ACGME: Accreditation Council for Graduate Medical Education
ASP: Academic Search Premier
BMC: BioMed Central Open Access
DOAJ: Directory of Open Access Journals
IO: intraoperative
JBI: Joanna Briggs Institute
Mini‐PAT: mini‐peer assessment tool
MSF: multisource feedback
OC: operative coaching
OR: operation room
PerO: perioperative
PGY: postgraduate year
PO: postoperative
PreO: preoperative
PRISMA‐ScR: Preferred Reporting Items for Systematic Reviews and Meta‐Analyses Extension for Scoping Reviews
ROAD: Directory of Open Access Scholarly Resources
SAP: Surgical Autonomy Programme
SEPAs: Surgical Entrustable Professional Activities
SIMPL: System for Improving and Measuring Procedural Learning
USA: United States of America
Wiley: Wiley–Blackwell

## 1 Introduction

### 1.1 Background

Feedback, conceptualised as information derived from direct observation, constitutes a pivotal instrument for learning and performance enhancement within educational contexts, particularly in medical and surgical training [1, 2]. While its precise underlying mechanisms remain somewhat elusive, its significance for resident physicians' training, ultimately aiming at the provision of highly qualified patient care, is well‐established [1].

Historically, the discourse surrounding feedback centred on optimising its delivery—addressing questions of quality, method and timing [3]. However, a notable paradigm shift has occurred, reorienting the focus towards understanding how learners actively receive and effectively utilise the information provided. This evolution has brought prominence to the concept of ‘feedforward,’ emphasising sustained learning progression and learner engagement [4, 5]. This shift underscores the critical importance of the learner's perspective, recognising that effective feedback transcends mere transmission, necessitating active reception and integration [6].

The residents' perception of and immediate reaction to feedback is thus as crucial as the chosen methodology or timing of its provision. Medical students and residents frequently report receiving insufficient feedback [7]. Furthermore, discrepancies in feedback perception often exist between residents and faculty members, although some studies indicate areas of shared understanding regarding effective feedback attributes [8]. Factors such as the teacher's credibility, the environment in which feedback is delivered, and the learner's individual experiences significantly influence this perception [2, 9]. Beyond external feedback, the internal, metacognitive feedback generated through self‐reflection is paramount for intentional learning and fostering a lifelong learner mindset [10].

Surgical residents acknowledge feedback as a source of valuable insights for future improvement, noting that its absence can lead to frustration and impact self‐confidence [11, 12]. They typically express a preference for immediate, face‐to‐face verbal feedback, ideally delivered during or directly following a surgical procedure, and value its prompt provision [13]. Nevertheless, it is commonly observed that postoperative (PO) feedback is delivered with less frequency than deemed optimal [14]. The existing scholarly literature on feedback in surgical education is characterised by methodological and contextual heterogeneity, impeding a cohesive and comprehensive understanding of surgical residents' perceptions. Scoping reviews are particularly well‐suited to synthesise evidence from such diverse fields [15]. A preliminary search indicates a notable scarcity of scoping reviews specifically addressing surgical residents' perceptions of feedback [16, 17], thereby highlighting a critical knowledge gap.

Against this backdrop, the present scoping review aims to systematically identify and map available data regarding surgical residents' perceptions of feedback received during their training, analyse these findings, delineate current knowledge gaps and disseminate the research outcomes.

## 2 Methods

This scoping review was meticulously designed and executed to ensure robust methodological rigour and transparency, adhering to best practices in evidence synthesis. It followed Arksey and O'Malley's (2005) five‐stage framework, encompassing the following: (i) identifying the research question, (ii) identifying relevant studies, (iii) selecting studies, (iv) charting the data and (v) collating, summarising and reporting the results. Consistent with the iterative nature of scoping reviews, minor refinements to the research question and inclusion criteria were made during the study selection and data charting phases to ensure optimal alignment with the emerging evidence. The review's structure aligns with The Joanna Briggs Institute's (JBI) scoping review methodology [18]. The guiding research question for this study was as follows: ‘What is known about surgical residents' perceptions of feedback in their training?’ The protocol for this scoping review has been previously published [19].

### 2.1 Search Strategy

The search strategy was conducted on March 15, 2024, by a specialised librarian. Key descriptors included the following: formative feedback, hospital medical staff, teaching, general surgery and perception, along with their Portuguese and Spanish equivalents, covering the period since 2017. Databases consulted were Medline (via PubMed), Directory of Open Access Journals (DOAJ), Directory of Open Access Scholarly Resources (ROAD), Academic Search Premier (ASP), BioMed Central Open Access (BMC) and Wiley–Blackwell (Wiley), using *Descritores em Ciências da Saúde* (*DeCS*) and Medical Subject Headings (MeSH) as appropriate. The deliberate exclusion of Scopus and Web of Science was based on evidence indicating substantial content overlap with the selected databases, yielding minimal additional relevant articles for considerable effort [20, 21]. This approach aligns with Preferred Reporting Items for Systematic Reviews and Meta‐Analyses Extension for Scoping Reviews (PRISMA‐ScR) guidelines [22, 23] and open science principles [24], while recognising that theoretical saturation is often achieved with four to six comprehensive databases [25, 26]. The complete search strategy is detailed in Appendix S1.

### 2.2 Inclusion and Exclusion Criteria

Studies were included if their population comprised resident physicians of any surgical specialty—defined broadly to include all surgical residency programmes such as general surgery, neurosurgery, ophthalmology, plastic surgery, cardiothoracic surgery, vascular surgery, urology, gynaecology and otorhinolaryngology—and if they addressed residents' perceptions of feedback. ‘Perceptions of feedback’ encompassed attitudes, experiences, values, beliefs, satisfaction and reported impact on learning and development. Eligible study types included qualitative and quantitative primary studies, systematic reviews, meta‐analyses, meta‐syntheses, books and guidelines, provided they directly focused on surgical residents' feedback perceptions. Publications such as opinions, consensuses, retractions, editorials, websites and advertisements were excluded due to their lack of empirical data on resident perceptions.

### 2.3 Study Selection

**FIGURE 1:**
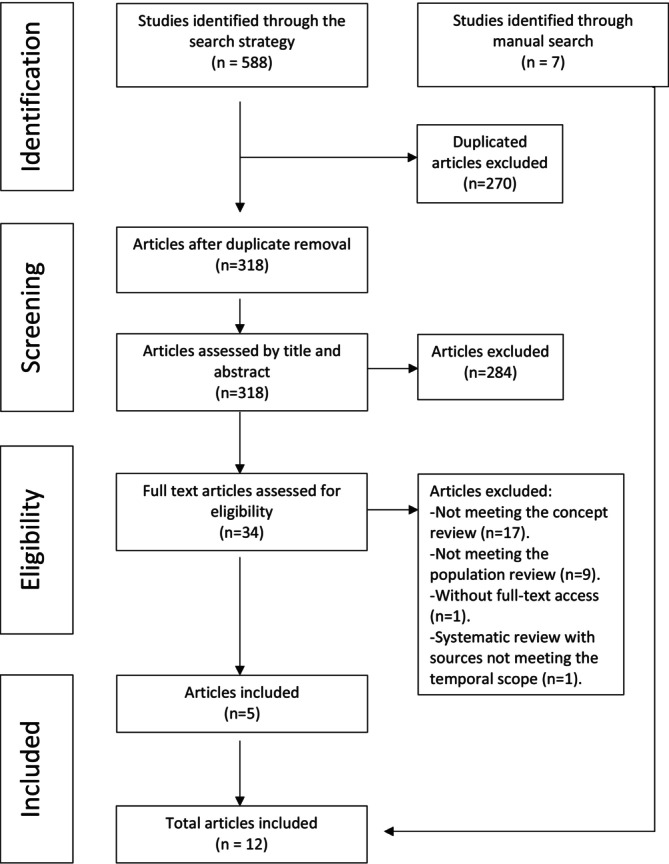
PRISMA‐ScR flowchart.

Following the exclusion of duplicate articles, titles and abstracts were independently screened by two researchers. An initial calibration exercise with 25 random articles achieved 92% agreement. Full texts of potentially relevant articles were then evaluated by the main researcher. Any disagreements during the selection process were resolved through discussion or by involving additional researchers. Reasons for full‐text exclusions are reported in the results. A manual search also led to the inclusion of additional eligible sources not identified by the initial strategy. The study selection process, aligned with PRISMA‐ScR guidelines [27, 28, 29, 30], is fully depicted in Figure 1. A list of included studies is available in Appendix S2 .

### 2.4 Charting the Data

Data from each selected study were charted by the main researcher using a Microsoft Excel spreadsheet. Recognising the iterative and flexible nature of scoping reviews [18, 27], the preliminary data extraction instrument outlined in the review protocol [19] was refined during the full‐text review phase to comprehensively capture the depth and heterogeneity of information. The refined instrument facilitated a detailed and systematic collection of variables. A comprehensive set of variables was extracted, encompassing study characteristics, feedback attributes (definition, method, delivery, learning domains, timing), residents' perceptions (positive, negative, influencing factors, effectiveness) and feedback's impact on education (learning, skill development, self‐confidence and reflection), alongside identified knowledge gaps, implications for practice and suggestions for future research. The complete data extraction tool is presented in Appendix S3.

### 2.5 Synthesis of Results

Both quantitative and qualitative analyses were performed on the extracted data. Quantitative descriptions and statistical analyses were applied to all data. For qualitative parameters, including feedback delivery methodology, learning domains, positive and negative aspects of perception, influencing factors and feedback's impact, all covered topics were systematically listed and then classified [31].

## 3 Results

The initial database search yielded 588 articles. The findings of each database are available in Appendix S4. Following a rigorous selection process, 270 duplicates were removed, and 284 articles were excluded after title and abstract screening. Of the 34 full‐text articles reviewed, five met the eligibility criteria. An additional seven articles were included through manual search, resulting in a total of twelve articles for comprehensive review. The reasons for exclusion were thoroughly documented, with common factors including population mismatch (e.g., nonsurgical residents or mixed groups without separate results), conceptual irrelevance (e.g., feedback in surgical education but not its perception) and full‐text unavailability. The entire study selection process is illustrated in Figure 1.

### 3.1 Characteristics of Included Studies

The 12 included studies were published between 2017 and 2024 (two in 2017, two in 2018, one in 2019, two in 2020, two in 2022, two in 2023 and one in 2024). The majority (83.4%; *n* = 10) were conducted in the United States of America, with single studies from Canada and Pakistan. All were primary studies, and the *Journal of Surgical Education* was the predominant publication source (nine studies). Methodological designs were diverse, comprising five mixed‐methods studies [32, 33, 34, 35, 36], five qualitative studies [12, 13, 37, 38, 39], one quantitative study [40] and one cross‐sectional survey [14]. Surgical specialties of resident participants predominantly included general surgery (nine studies), alongside ophthalmology [32], neurosurgery [34], plastic surgery [13, 38, 39], thoracic surgery [38] and vascular surgery [35, 38]. Postgraduate year (PGY) levels varied, and sample sizes ranged from 7 participants [12] to 83 respondents [40], with some studies analysing a large number of evaluations (e.g., 2968 entries in Neal et al. [34]). Academic medical centres and university hospitals constituted the primary institutional contexts. A detailed overview is provided in Table 1.

**TABLE 1:** General data of the articles.

Authors	Year	Country	Journal	Design	Residents' specialty	Sample size
Kamal et al. [32]	2017	Pakistan	Pakistan Armed Forces Medical Journal	Mixed (prospective)	Ophtalmology	10 residents
Nathwani et al. [14]	2017	USA^a^	Journal of Surgical Education	Cross‐sectional survey (mixed)	General surgery	23 residents
Bello et al. [13]	2018	USA	Journal of Surgical Education	Qualitative	General and plastic surgery	23 residents +7 fellows
Lees et al. [12]	2018	Canada	Journal of Surgical Education	Qualitative	General surgery	7 residents
Dedhia et al. [33]	2019	USA	Journal of Surgical Education	Mixed (prospective)	General surgery	23 residents
Gupta et al. [40]	2020	USA	Journal of Surgical Education	Cross‐sectional survey (quantitative)	General surgery	83 residents
Vu et al. [37]	2020	USA	Journal of Surgical Education	Qualitative	General surgery	18 residents
Neal et al. [34]	2022	USA	Journal of Surgical Education	Embedded mixed methods (prospective)	Neurosurgery	42 residents (2968 evaluations)
Rivard et al. [38]	2022	USA	Journal of Surgical Education	Qualitative (retrospective)	General, plastic, thoracic, vascular surgery	46 residents (355 evaluations)
Sisak et al. [35]	2023	USA	The American Journal of Surgery	Mixed (retrospective, cross‐sectional)	Surgical subspecialties	50 residents
Collings et al. [39]	2023	USA	Academic Medicine	Qualitative	General surgery	39 residents
Go et al. [36]	2024	USA	Journal of Surgical Education	Mixed (prospective)	General surgery	22 chief residents (441 evaluations)

^a^ United States of America.

### 3.2 Feedback Characteristics

#### 3.2.1 Feedback Concept, Method and Delivery Methodology

Most studies did not explicitly define ‘feedback’; when mentioned, it generally aligned with ‘information about a learner's performance intended to improve performance.’ Only Kamal et al. (2017) explicitly mentioned multisource feedback (MSF) and learning conversation methods. Feedback delivery methodologies varied considerably, including: MSF or 360° feedback [32], face‐to‐face and one‐on‐one feedback sessions [13, 32], immediate verbal feedback (often preferred in the operating room [OR] or immediately post‐operatively) [13, 14, 33, 39, 40], formal (written) [12, 37], nonverbal interactions [33], delivery via online tools (such as Surgical Autonomy Programme [SAP] and Surgical Entrustable Professional Activities [SEPAs] evaluations) [34, 36], faculty evaluation comments [38] and structured PO debriefings [39].

Instrumentation for feedback was frequently unspecified (4/12 articles), characterised as formal/informal (3/12 each), or via specific written instruments (3/12).

#### 3.2.2 Feedback Learning Domains

Feedback explicitly addressed diverse learning domains including: technical skills and operative performance [12, 13, 33, 34, 36, 39, 40], nontechnical skills (communication, interpersonal skills, collegiality, humanism, professionalism, resource utilisation and reliability) [13, 14, 32], clinical judgement and decision‐making [13, 33, 39, 40], leadership [37], autonomy and progression towards independence [34, 36, 38] and self‐regulated learning and reflection [36].

#### 3.2.3 Feedback Timing

Most studies (75%; *n* = 9) focused on feedback provided during the perioperative (PerO) period, with PO feedback being the most prevalent. Other articles referred to feedback delivered at any point during residency. One study noted monthly and semi‐annual formal evaluations [37]. Table 2 provides additional details.

**TABLE 2:** Articles' feedback data.

Authors	Feedback method	Feedback delivery methodology addressed	Feedback timing	Learning domains focused
Kamal et al. [32]	MSF^a^, learning conversation	MSF (mini‐PAT^b^), face‐to‐face sessions, confidentiality	Anytime	Attitude, communication, interpersonal, professionalism
Nathwani et al. [14]	Not mentioned	Not mentioned	PO^c^	Technical skills, nontechnical skills (communication, teamwork, leadership)
Bello et al. [13]	Not mentioned	Verbal, face‐to‐face, during/immediately after case	PO	Operative performance, technical skills, clinical judgement
Lees et al. [12]	Not mentioned	Informal (verbal), formal (written)	IO^d^, PO	Operative performance, technical skills, clinical judgement, errors
Dedhia et al. [33]	Not mentioned	Verbal and nonverbal, immediate (PO preference)	IO and PerO^e^	Technical skills, anatomical knowledge, clinical judgement
Gupta et al. [40]	Not mentioned	Not mentioned	PreO^f^, IO, PO	PerO care, operative performance, judgement, identifying improvements
Vu et al. [37]	Not mentioned	Formal (evaluations), informal (in‐person, on‐the‐fly, cues)	Anytime, monthly end‐of‐rotation evaluations, semiannual review	Leadership development, emotional intelligence, conflict management
Neal et al. [34]	Not mentioned	Written (via SAP^g^), ZPD^h^ and TAGS^i^ context	PO	Operative skills, progression towards independence, identifying deficiencies
Rivard et al. [38]	Not mentioned	Verbal (direct, practical, granular), implicit	PreO, IO, PO	Operative skills, autonomy/entrustment
Sisak et al. [35]	Not mentioned	Not mentioned	Anytime	Not specified, but related to general learning on rotations
Collings et al. [39]	Not mentioned	Not mentioned	IO, PO	Technical skills, clinical judgement, identifying deficiencies
Go et al. [36]	Not mentioned	Verbal and/or written SEPA^j^‐based	PO	Operative skills, autonomy, entrustment, self‐regulated learning, ergonomics

^a^ Multisource feedback

^b^ Peer Assessment Tool

^c^ Postoperative

^d^ Intraoperative

^e^ Perioperative

^f^ Preoperative

^g^ Surgical Autonomy Program

^h^ Zone of Proximal Development

^i^ Teach and demonstrate, Advise and scaffold, Guide and monitor, Solo and observe

^j^ Surgical Entrustable Professional Activity

### 3.3 Residents' Perceptions of Feedback

#### 3.3.1 Positive Aspects of Perception

Residents consistently perceived feedback as highly valuable and important for development and learning [12, 13, 14, 35, 40]. It was also reported as beneficial for learning and satisfaction with rotations [35], confidence‐boosting (particularly positive feedback) [12, 36], more constructive and valuable when delivered by senior residents [40] and effective for improving self‐regulated learning and awareness of strengths/weaknesses [36]. Feedback was generally well received [32], preferable when verbal and face‐to‐face (ideally during or immediately after a surgical case) [13, 14] and desired with greater frequency, especially with dedicated and structured time allocation [14, 40].

#### 3.3.2 Negative Aspects of Perception

Conversely, residents reported concerns including the low frequency of feedback (particularly outside the OR) [14, 40], lack of specificity, clarity, or explicit labelling as ‘feedback’ [33, 34, 40] and delayed delivery, leading to perceived irrelevance [13, 37]. Feedback was perceived negatively when delivered in an inadequate environment without privacy [14], without guidance for improvement (which can be unhelpful and detrimental to confidence) [12, 39] and when accompanied by detrimental comments (demeaning, sarcastic, personal attacks) [38, 39]. Residents also noticed variability in feedback quality among attendings [12, 34] and that attendings sometimes failed to remember resident performance [34]. Written feedback was perceived as less valuable compared to verbal feedback [40].

*Feedback was perceived negatively when delivered in an inadequate environment without privacy*.

#### 3.3.3 Factors Influencing Perception

Several factors were identified as shaping residents' perceptions. Time constraints and competing attending responsibilities affect feedback frequency and quality [13, 14]. When delivered in calm, respectful and collaborative settings, feedback is perceived more positively than that delivered in hostile environments [38, 39, 40]. Senior residents are often perceived as providing more constructive feedback than attending surgeons [40]; with attending personal traits such as kindness, patience, calmness, approachability and respect serving as factors that positively influence perception [38, 39]. The rapport between residents and attendings is reported as crucial [12, 38] in determining feedback quality. Explicit, direct, practical, granular and specific feedback is perceived as higher quality [33, 34, 36, 38, 39]. Immediate verbal PO feedback is considered ideal, while written and delayed feedback is less valued [13, 14]. It was also reported that perceptions may vary between junior and senior residents [13] and the presence or absence of an institutional ‘feedback culture’ impacts both provision and reception [37, 40].

### 3.4 Other Specific Aspects of Perception

#### 3.4.1 Comparison of Perceptions (Residents vs. Preceptors)

No consistent correlation was found between residents' and preceptors' perceptions regarding feedback frequency or quality. Nathwani et al. (2017) reported differing ideal practices, while Dedhia et al. (2019) noted agreement on timing and subjective areas, but discrepancies in perceived frequency.

No consistent correlation was found between residents' and preceptors' perceptions regarding feedback frequency or quality.

#### 3.4.2 Immediate Reaction to Feedback

Only Kamal et al. (2017) described residents' immediate reactions, characterising behaviours as friendly, highlighting a gap in the literature.

#### 3.4.3 Data Collection Methodologies for Feedback Perception

Studies employed diverse methods to investigate perceptions. Surveys, questionnaires [14, 32, 33, 35, 40], semistructured interviews and focus groups [12, 13, 36, 37, 38, 39] were the most prevalent. Video analysis of perceived events [33] and text analysis of comments and evaluations [34, 36, 38] were also utilised.

Table 3 details these findings.

**TABLE 3:** Key aspects of residents' perception of feedback and data collection methods for perception.

Authors	Key findings on residents' perception	Positive aspects of perception	Negative aspects of perception	Factors influencing perception	Data collection method for perception
Kamal et al. [32]	Overall improvement in residents' attitudes, feedback accepted amicably, correlation between self‐scoring and other raters' scoring.	Feedback accepted amicably; positive personality/skill traits.	One resident denied smoking when confronted, another reacted aggressively. Failure to ensure secrecy leads to rivalry.	Accurate and timely delivery, confidentiality, facilitative feedback sessions, credibility of sources.	Modified mini‐PAT^a^ questionnaire and semi‐structured interviews
Nathwani et al. [14]	PO^b^ feedback highly valued, but received significantly less frequently than desired.	PO feedback has high educational value; strong desire for increased frequency; preference for immediate verbal in OR^c^.	Significant discrepancy between current and desired frequency; substantial barriers (time, competing responsibilities, privacy, perceived lack of interest).	Time, competing responsibilities, inappropriate environment, perceived lack of interest from attending.	Survey with open‐ended and closed questions
Bello et al. [13]	Operative performance feedback very important; preference for verbal, face‐to‐face, immediate; variability in quality/timeliness.	High importance; verbal, face‐to‐face most valuable; immediate or within 1 week is preferred.	Delayed feedback; performance rating tools do not replace verbal; variability in quality/timeliness.	Type of feedback, timing of delivery, quality of feedback, attending characteristics, level of training.	Semi‐structured interviews
Lees et al. [12]	Feedback is important for confidence; direct, useful, constructive feedback is valued; variability among attendings.	High importance for development; positive reinforcement; constructive guidance with explanation.	Feedback without guidance for improvement; negative impact on confidence; variability in quality; poor staff‐resident relationship.	Content and constructiveness, formality, source (relationship with attending), timing (post‐task).	Semi‐structured interviews
Dedhia et al. [33]	Residents perceive IO^d^ feedback with less frequency/specificity/immediacy than faculty; similar overall satisfaction.	Overall satisfaction with operative feedback was similar to faculty.	Lower frequency, specificity and immediacy; less identification of nonverbal interactions as feedback; preference for post‐operative feedback.	Position (resident vs. faculty), explicitness/labelling, nonverbal cues, interpretation (feedback vs. teaching), timing preference.	Video timestamping, survey (Likert scale & free text comments)
Gupta et al. [40]	Feedback important but low frequency; preference for structured time; senior resident feedback more constructive.	Generally helpful and useful (especially from senior residents); strong desire for dedicated, structured time PreO^e^/PO.	Overall low frequency; particularly lacking intraoperatively; attendings rarely explicit/create safe environment.	Timing/PerO^f^ phase, institution type, feedback provider, explicitness, learning environment, format/structure, culture.	Electronic survey (Likert scale questions)
Vu et al. [37]	Residents highly value leadership‐specific feedback, but current mechanisms are inadequate; structural and personal barriers.	Very important for development; timely in‐person feedback is valuable.	Formal (delayed, nonspecific, not leadership‐focused); informal (difficult to recognise); cultural/personal barriers.	Source, timing, specificity, focus, delivery method, relationship/rapport, environment/culture, personal factors (receiver/giver).	Semi‐structured interviews
Neal et al. [34]	Perceived quality of written feedback based on content (specific, key points, independence) and faculty's approach.	Specific to performance, key learning points, progress towards independence; use of ZPD^g^; any written comment is better than blank; timely.	Nonspecific, blank; not remembering resident's performance is highly detrimental; focus solely on ‘next steps’ not significantly better; longer completion time.	Content specificity, absence of content, explicit lack of recall, faculty's use of ZPD, timeliness of faculty completion, social contract/relationship.	Quality categorisation within SAP^h^ system, report of ZPD use
Rivard et al. [38]	Faculty's effectiveness in promoting development influenced by personal traits, learning environment and teaching techniques.	Positive traits (kind, patient); supportive environment (calm, teamwork); effective techniques (goal setting, debrief, constructive feedback, appropriate autonomy).	Negative traits (condescending, unpredictable); stressful environment (hostile); ineffective techniques (micromanaging, observer role, sarcastic).	Attending's personal traits, learning environment, teaching techniques, communication skills, relationship/rapport, attending's focus.	Qualitative analysis of open‐ended comments in faculty evaluations
Sisak et al. [35]	Subspecialty residents perceive appropriate feedback from general surgery attendings as beneficial to learning.	Receiving ‘appropriate feedback’ is ‘beneficial to their learning’ (83% agreement); linked to higher overall satisfaction.	Not mentioned.	Receiving feedback, feedback provider (general surgery attendings), association with overall rotation satisfaction.	Survey (Likert scale)
Collings et al. [39]	Effective IO teaching and feedback depend on expertise, attending's character, instructional approach and ability to discern resident needs.	Actionable, constructive (even critical), timely (IO for technical, PO for overall); valued debriefing; affirmative feedback; variety of teachers.	Demeaning/personal attacks; lack of constructiveness; vague language; lack of caring/respect; lack of self‐control/unpredictability; lack of trust in attending's skill; taking over without explanation.	Teacher's character, teacher's IO skill, instructional approach, feedback content and delivery, discernment of resident needs, variety of teachers, relationship/environment.	Semi‐structured focus groups
Go et al. [36]	OC programme and feedback effective in improving self‐regulated learning and promoting confidence/autonomy.	Effectiveness (confidence, autonomy); valued neutral authentic feedback, third‐party observation, actionable feedback; specificity and usefulness.	Underestimate their own skills; attendings overestimate autonomy granted; potential biases (gender, racial).	Source of observation, feedback characteristics, SEPAs^j^ evaluation, relationship with evaluators, self‐reflection tendency, attending behaviour.	Qualitative interviews, qualitative text analysis, quantitative comparison of evaluations

^a^ Peer Assessment Tool

^b^ Postoperative

^c^ Operation room

^d^ Intraoperative

^e^ Preoperative

^f^ Perioperative

^g^ Zone of Proximal Development

^h^ Surgical Autonomy Program

^i^ Operative coaching

^j^ Surgical Entrustable Professional Activities

#### 3.4.4 Impact of Feedback on Resident's Education

Few studies directly measured the impact of feedback, with most reporting general perceptions. However, some key impacts were identified:

#### 3.4.5 Impact on Learning Progress

Kamal et al. reported overall improvement in residents' attitudes and practice, with statistically significant increases in scores post‐intervention and specific behavioural changes [32]. Improved self‐regulated learning, awareness of strengths/weaknesses and progression of skills and autonomy throughout residency were reported by Go et al. [36].

#### 3.4.6 Impact on Skill Development

This was the most prevalent impact reported, including: development of nontechnical skills [32], enhancement of operative skills and autonomy [36, 38] and improvement in judgement and decision‐making, especially in PO debriefings [39, 40].

#### 3.4.7 Impact on Self‐Confidence

Self‐confidence was positively impacted by positive feedback [12] and operative coaching (OC) programmes [36].

#### 3.4.8 Impact on Reflection/Internal Feedback

Go et al. reported that feedback facilitates reflection, self‐regulated learning, helping residents identify strengths and weaknesses for internal reflection and goal setting [36].

Table 4 summarises the impact of feedback.

**TABLE 4:** Impact of feedback on residents' education.

Authors	Impact on learning progress	Impact on skill development	Impact on self‐confidence	Impact on reflection/internal feedback
Kamal et al. [32]	Overall improvement in attitudes and practice; behavioural changes (reduced smoking, increased study time).	Development of communication, interpersonal skills, collegiality, humanism, professionalism.	Not mentioned.	Not mentioned.
Nathwani et al. [14]	Not mentioned.	Not mentioned.	Not mentioned.	Not mentioned.
Bello et al. [13]	Not mentioned.	Not mentioned.	Not mentioned.	Not mentioned.
Lees et al. [12]	Not mentioned.	Not mentioned.	Positive feedback significantly boosts confidence.	Not mentioned.
Dedhia et al. [33]	Not mentioned.	Not mentioned.	Not mentioned.	Not mentioned.
Gupta et al. [40]	Not mentioned.	Improvement IO^a^/operative performance; judgement/decision‐making.	Not mentioned.	Not mentioned.
Vu et al. [37]	Not mentioned.	Not mentioned.	Not mentioned.	Not mentioned.
Neal et al. [34]	Not mentioned.	Improvement IO performance/technical ability; progression towards independence.	Not mentioned.	Not mentioned.
Rivard et al. [38]	Not mentioned.	Development of operative skills and progression of autonomy/entrustment.	Not mentioned.	Not mentioned.
Sisak et al. [35]	Not mentioned.	Not mentioned.	Not mentioned.	Not mentioned.
Collings et al. [39]	Not mentioned.	Improvement in technical skills and clinical judgement.	Not mentioned.	Not mentioned.
Go et al. [36]	Improved self‐regulated learning; skill and autonomy progression (SEPAs^b^).	Improved operative skills (PSS^c^, GS^d^); autonomy (SSG^e^).	OC^f^ programme promotes confidence.	Facilitates reflection and self‐regulated learning; helps gain awareness of strengths/weaknesses.

^a^ Intraoperative.

^b^ Surgical Entrustable Professional Activities.

^c^ Procedural‐specific skill.

^d^ General skill.

^e^ Step‐specific guidance.

^f^ Operative coaching.

### 3.5 Knowledge Gaps and Implications

#### 3.5.1 Knowledge Gaps Identified by the Study Authors

The two most prevalent gaps were the absence of attending perspectives, which can limit understanding [12, 13, 34, 37, 38], and the prevalence of single‐centre studies, which affect generalizability [12, 13, 34, 35, 36, 37, 38, 39, 40]. Other key gaps included a lack of consensus between faculty and trainees regarding learning needs and high‐quality feedback components [13], challenges in consistent feedback delivery [14], limited research on leadership‐specific feedback [37], insufficient understanding of operative skill and entrustment progression in coaching programmes [36], unclear reasons for feedback frequency differences between university and community hospitals [40] and uncertainty regarding whether Accreditation Council for Graduate Medical Education (ACGME) assessments provide adequate feedback for leadership development [37].

#### 3.5.2 Implications for Practice

The review's findings suggest attendings should prioritise feedback in preoperative (PreO) and PO phases [40], institute dedicated and structured feedback sessions [40] and modify PO workflow to incorporate teaching moments [14]. Similarly, institutions should develop faculty programmes for effective feedback delivery, emphasising explicit labelling and safe environments [34, 38, 39, 40], implement coaching programmes and structured assessment tools like SEPAs [36], focus on attendings' personal traits and the learning environment to improve operative performance and autonomy [38, 39] and promote a broader ‘feedback culture’ [37, 40]. Feedback from senior residents should also be leveraged [40].

#### 3.5.3 Suggestions for Future Research by the Study Authors

Following the identified gaps, the study authors suggest that larger and multicentre studies be conducted to improve generalizability [12, 13, 14, 34, 35, 36, 37, 38, 39, 40] and that faculty and other team members' perspectives be included [12, 13, 34, 37, 38, 39]. They also suggest that future research should evaluate the impact of interventions designed to improve feedback [13, 14, 37, 40], investigate biases in evaluations, such as those related to gender and race [36], explore why residents underestimate skills while attendings overestimate autonomy [36], research more effective methods for teaching and providing feedback [40] and study verbal feedback during operative cases [34].

Table 5 provides a comprehensive summary of these aspects. The complete tables are available in Appendix S5.

**TABLE 5:** Knowledge gaps, implications for practice and suggestions for future research.

Authors	Knowledge gaps identified by the study authors	Implications for practice	Suggestions for future research by the study authors
Kamal et al. [32]	Little research on MSF^a^ in Pakistani context.	MSF can improve practice; confidentiality is crucial.	Study role of MSF in Pakistani doctors; MSF for faculty.
Nathwani et al. [14]	Little known on consistent PO^b^ feedback delivery; validating perceptions.	PO feedback is crucial; modify workflow; cultural shift.	Validate resident/attending perceptions; multi‐institutional studies; qualitative methods for ideal feedback.
Bello et al. [13]	Limited evidence on best feedback delivery; lack of consensus between faculty/trainees.	Improve operative training; incorporate trainee perspective; optimise performance rating tools.	Preceptor perspective; larger/more targeted samples; subgroup comparisons; intervention studies.
Lees et al. [12]	Factors influencing confidence; ‘confidence crisis’ in residents.	Improve learning experiences; accelerate progress; constructive feedback; cultivate supportive environments.	Replicate with larger sample; include PGY^c^ 1 and 5; preceptor perspective; impact of gender.
Dedhia et al. [33]	Characterise feedback types (nonverbal); satisfaction despite lower frequency.	Explicitly label feedback; PO review; use structured models; video review as a tool.	Understand resident satisfaction; delineate reasons for not identifying interactions; larger video studies.
Gupta et al. [40]	Reasons for differences between university/community hospitals; congruence resident/faculty perceptions; faculty comfort.	Prioritise PreO^d^/PO feedback; structured time; faculty development; leverage senior resident feedback; safe environment.	Survey faculty; larger, broader studies; reasons for hospital differences; congruence of perceptions.
Vu et al. [37]	How leadership feedback is delivered; if ACGME^e^ assessments are adequate.	Implement formal leadership assessment; overcome barriers; promote positive environment; coaching model.	Faculty/team member perspectives; in‐depth leadership feedback; multiprogramme studies; intervention studies.
Neal et al. [34]	Only written feedback evaluated; no follow‐up with faculty/residents; no common frame of reference.	Prioritise specific comments (performance, key points); avoid blank/nonspecific comments; avoid lack of recall.	In‐depth analysis of feedback value; faculty perspective; verbal feedback; impact of faculty‐resident relationship.
Rivard et al. [38]	Single‐centre study; sampling limitations; subjectivity/biases in evaluation.	Focus on personal traits/environment; faculty development programmes; supportive environment; mentorship.	Larger/multi‐institutional samples; nuances of faculty behaviours; other OR^f^ team members' perspectives; impact of interventions.
Sisak et al. [35]	Limited data on surgical subspecialty residents in general surgery rotations; reasons for lower case volume.	Core surgical rotations are important; focus on learning opportunities (not just volume); value feedback and mentorship.	Investigate case discrepancy; explore why residents do not log cases; evaluate interventions (defined curricula).
Collings et al. [39]	Learner perspective less explored; difference between faculty/resident assessment; single‐centre study.	Emphasise teacher's character; tangible feedback strategies; timely debriefing; individualise instruction; celebrate diverse styles.	Compare junior/senior resident perceptions; faculty interviews; multi‐institutional studies; investigate coaching strategies.
Go et al. [36]	Little known about skill/entrustment progression in coaching; what specific OC^g^ elements are effective.	Implement SEPAs^h^/coaching; focus on autonomy; enhance teaching/learning; adjust teaching plans; address self‐efficacy deficits.	Larger qualitative samples; gender/racial biases; compare OC outcomes vs. traditional methods; implementation hurdles.

^a^ Multisource feedback.

^b^ Postoperative.

^c^ Postgraduate year.

^d^ Preoperative.

^e^ Accreditation Council for Graduate Medical Education.

^f^ Operation room.

^g^ Operative coaching.

^h^ Surgical Entrustable Professional Activities.

## 4 Discussion

This scoping review aimed to map the current understanding of surgical residents' perceptions of feedback, identifying key characteristics and literature gaps. Our findings highlight persistent challenges in understanding this crucial aspect of medical education [1, 41].

A significant observation was the scarcity and methodological heterogeneity of included articles. Despite a broad search, most studies were single‐centre, predominantly from the United States, and primarily retrospective. This methodological diversity, encompassing varied designs and assessment approaches, hinders direct comparisons and robust evidence synthesis [42]. The over‐reliance on retrospective data introduces recall bias, potentially compromising the accuracy of reported perceptions [43], contrasting with the more accurate capture offered by prospective studies [44]. The higher number of manually included articles compared to those from the search strategy also suggests potential limitations in current search practices for this evolving field [28].

The resident sample heterogeneity, particularly in PGY levels and sample sizes (e.g., Lees et al., 2019), further complicates generalizability, as perceptions vary significantly with training progression. Notably, most studies did not explicitly define ‘feedback’ for participants, potentially affecting the consistency and interpretation of resident perceptions.

Feedback methods were rarely detailed, with verbal and written forms being most common, and specific instruments used less frequently. This lack of standardisation makes it difficult to ascertain effective approaches. While residents prefer timely, direct and explicit feedback [13, 14], the ‘how’ of feedback delivery (e.g., specific models like Pendleton) is often missing, impeding the identification of best practices [2]. The prevalent PerO feedback, especially PO, often delayed, contrasts with the recognised importance of PreO and intraoperative (IO) feedback. The perceived lack of time by preceptors to deliver quality feedback highlights a systemic barrier requiring institutional solutions [7].

Feedback content covered diverse learning domains—cognitive, affective and psychomotor—though rarely all simultaneously. Psychomotor skills, particularly operative performance and technical skills, were most prevalent, reflecting the practical nature of surgical training. However, this emphasis underscores the need for more holistic feedback approaches.

Residents consistently value feedback as crucial for their development and confidence [12, 13], especially when positive. Key negative perceptions include low frequency [14], lack of specificity or explicit labelling [33] and delayed delivery [13]. Factors influencing perception include timing, learning environment, source (senior residents often preferred), explicitness, attending traits and institutional culture [40]. Discrepancies between residents' and preceptors' perceptions are common, with residents often under‐recognising feedback that preceptors believe they provide [8]. This communication gap suggests a need for improved delivery techniques and a shared understanding of effective feedback [45].

Many words were used by the residents to describe the feedback effectiveness, but the most common were ‘useful’, ‘direct’, ‘formative’, ‘regular’ and ‘specific’. In a general way, feedback was perceived as more effective by residents when delivered postoperatively (‘at the end of the OR’), both verbally and in writing. Nevertheless, when in writing, residents demonstrated preference if it was made using a standard instrument. Some of the instruments used for this purpose were SEPAs evaluations [36], SAP online assessment tool [34], OpTrust scores [38] and mini‐peer assessment tool (mini‐PAT) questionnaire [32]. Though already described in literature, the use of an App‐based system for feedback delivery like System for Improving and Measuring Procedural Learning (SIMPL) [46, 47] and Zwisch Me!! [48] was not reported by the studies included in this scoping review. As already demonstrated by Steinhagen et al. [49], the use of an online feedback tool does not necessarily mean an improvement in feedback effectiveness. However, perhaps more than the utilisation of a standardised instrument, an App‐based system, or an online feedback tool, the feedback effectiveness increases if the feedback is explicit, objective and direct, which is surely easier to do with one of these instruments.

While many words describe feedback effectiveness, its actual impact was seldom measured. Only Kamal et al. (2017) and Go et al. (2024) prospectively assessed impact on learning and skill development, highlighting improvements in attitudes, self‐regulated learning and autonomy. Critically, the impact on patient care quality or learner engagement was largely underexplored, representing a significant gap given feedback's ultimate goal [7]. This deficiency underscores a need to move beyond mere satisfaction to demonstrable behavioural change and improved performance—the ‘feedforward’ concept [4]. The absence of standardised, validated instruments for assessing resident perceptions remains a methodological limitation [50].

Identified knowledge gaps point to crucial research directions: the need for larger, multicentre studies [12], inclusion of faculty perspectives [12], evaluation of feedback interventions [13] and investigation of biases [36], exploration of technology‐enhanced feedback [49] and investigation of different feedback methods/instruments' effectiveness.

## 5 Limitation

This review has important limitations. Firstly, the search strategy was limited to English, Portuguese and Spanish, excluding articles from other languages and cultures. Given that feedback conceptions and practices vary culturally [51], the findings may not universally represent global feedback perceptions. Secondly, consistent with Arksey and O'Malley's (2005) framework, stakeholder consultation was not undertaken. While the review comprehensively maps the literature, engaging surgical residents, preceptors and experts could have offered additional perspectives and validated interpretations of identified gaps and opportunities. Thirdly, the inherent methodological heterogeneity of the included studies, particularly the predominance of retrospective designs and varied sample sizes, limits the ability to draw definitive conclusions and conduct rigorous comparisons.

## 6 Conclusions

This scoping review reveals that while feedback is universally acknowledged as crucial for learning and skill development in surgical education, the current evidence base presents significant limitations. There is a pervasive lack of standardised methods and instruments for both providing and assessing feedback, leading to heterogeneous data and comparison challenges. A critical underemphasis exists on directly measuring the actual impact of feedback on residents' learning outcomes and on the ‘feedforward’ concept of actionable improvement.

*Critical underemphasis exists on directly measuring the actual impact of feedback on residents' learning outcomes*.

The review also highlights existing discrepancies between residents' and preceptors' perceptions, insufficient detail in describing feedback characteristics and a scarcity of prospective studies, which limits the depth of understanding regarding immediate reactions and the evolution of perceptions over time. Furthermore, the limited cultural diversity in existing literature suggests that current findings may not fully represent global feedback perceptions in surgical education. In summary, this review underscores a critical need for more standardised, comprehensive and impact‐focused research to truly understand and enhance feedback practices in surgical training, ultimately contributing to improved patient care.

*This review underscores a critical need for more standardised, comprehensive and impact‐focused research*.

## Author Contributions

**Carlos Dario da Silva Costa:** conceptualization, data curation, investigation, formal analysis, writing – original draft, writing – review and editing, project administration, methodology. **Gabriela Gouvea Silva:** investigation, data curation. **Emerson Roberto dos Santos:** writing – review and editing. **Alba Regina de Abreu Lima:** writing – review and editing. **Vânia Maria Sabadoto Brienze:** writing – review and editing. **Denise Cristina Móz Vaz Oliani:** writing – review and editing. **Antonio Hélio Oliani:** writing – review and editing. **Júlio César André:** conceptualization, formal analysis, writing – review and editing, supervision. All authors contributed to the manuscript reading and the approval of the submitted version.

## Funding

This study received funding from the Coordenação de Aperfeiçoamento de Pessoal de Nível Superior (CAPES).

## Ethics Statement

The authors have nothing to report.

## Consent

The authors have nothing to report.

## Conflicts of Interest

The authors declare no conflicts of interest.

## Permission to Reproduce Material From Other Sources

Not applicable.

## Supporting information


**Appendix S1:** Descriptors used as search strategy.

**Appendix S2:** List of included studies and their characteristics.

**Appendix S3:** Data extraction tool.

**Appendix S4:** Search strategies and results for all databases searched.

**Appendix S5:** Tables summarising the results.

## Data Availability

The data that support the findings of this study are available in the Supporting Information of this article.
