# Supplementary material for: Surgical Residents' Feedback Perceptions: A Scoping Review on Gaps and Improvements
Source: Clin Teach. 2025 Dec 15;23(1):e70323. doi: 10.1111/tct.70323 (PMC12706175; doi:10.1111/tct.70323)
Supplement: Supplementary file 1 — Appendix S1: Descriptors used as search strategy. [file TCT-23-e70323-s004.docx]

Appendix 1: Descriptors used as search strategy.

| SUBJECTS | SUBJECT AND SYNONYMS IN  PORTUGUESE (DeCS) | SUBJECT AND SYNONYMS IN ENGLISH (MeSH) | SUBJECT AND SYNONYMS IN  SPANISH (DeCS)* |
| --- | --- | --- | --- |
| SUBJECT 1 | “Feedback Formativo” | *“Formative Feedback” OR “Feedback, Formative” OR “Constructive Feedback” OR “Feedback, Constructive” OR “Feedback (Learning)”* | *“Retroalimentación Formativa”* |
| SUBJECT 2 | ““Corpo Clínico Hospitalar” *OR* “Chefe de Serviços Médicos Hospitalares” *OR* “Corpo Médico Hospitalar” *OR* “Médico Residente” *OR* “Médicos Recém-Formados” *OR* “Médicos Residentes” | *“Medical Staff, Hospital” OR “Hospital Medical Staff” OR “Hospital Medical Staffs” OR “Staff, Hospital Medical” OR “Staffs, Hospital Medical” OR “Medical Staffs, Hospital” OR “Physicians, Junior” OR “Junior Physician” OR “Junior Physicians” OR “Physician, Junior” OR “Registrars, Hospital” OR “Hospital Registrar” OR “Hospital Registrars” OR “Registrar, Hospital” OR “Attending Physicians, Hospital” OR “Hospital Attending Physician” OR “Hospital Attending Physicians” OR “Attending Physician, Hospital”* | *“Cuerpo Médico de Hospitales”* |
| SUBJECT 3 | “Ensino” *OR* “Atividade de Treinamento” “Atividades Formativas” *OR* “Atividades de Capacitação” *OR* “Atividades de Formação” *OR* “Atividades de Treinamento” *OR* “Atividades de Treino” *OR* “Capacitação Acadêmica” *OR* “Didática” *OR* “Docência” *OR* “Formação Acadêmica” *OR* “Método de Ensino” *OR* “Métodos Pedagógicos” *OR* “Métodos de Ensino” *OR* “Pedagogia” *OR* “Treinamento Acadêmica” *OR* “Treino Acadêmico” *OR* “Técnica de Treinamento” *OR* “Técnicas Educacionais” *OR*  “Técnicas Educativas” *OR* “Técnicas de Ensino” *OR* “Técnicas de Formação” *OR* “Técnicas de Treinamento” *OR* “Técnicas de Treino” | *“Teaching” OR “Training Techniques” OR “Training Technique” OR “Technique, Training” OR “Techniques, Training” OR “Training Technics” OR “Technic, Training” OR “Technics, Training” OR “Training Technic” OR “Pedagogy” OR “Pedagogies” OR “Teaching Methods” OR “Teaching Method” OR “Method, Teaching” OR “Methods, Teaching” OR “Academic Training” OR “Training, Academic” OR “Training Activities” OR “Training Activity” OR “Activities, Training” OR “Activity, Training” OR “Techniques, Educational” OR “Educational Techniques” OR “Educational Technique” OR “Technique, Educational” OR “Educational Technics” OR “Educational Technic” OR “Technic, Educational” OR “Technics, Educational”* | *“Enseñanza”* |
| SUBJECT 4 | “Cirurgia Geral” | *“General Surgery” OR “Surgery, General” OR “Surgery”* | *“Cirugía General”* |
| SUBJECT 5 | “Percepção” | *“Perception”* | *“Percepción”* |
