# Supplementary material for: Surgical Residents' Feedback Perceptions: A Scoping Review on Gaps and Improvements
Source: Clin Teach. 2025 Dec 15;23(1):e70323. doi: 10.1111/tct.70323 (PMC12706175; doi:10.1111/tct.70323)
Supplement: Supplementary file 2 — Appendix S2: List of included studies and their characteristics. [file TCT-23-e70323-s001.docx]

Appendix 2: List of included studies and their characteristics

| **1. Kamal, Z., Iqbal, U., Akhlaq, S., Adil, A., & Ramzan, M. (2017). Does use of multi-source feedback (MSF) affect the attitudes of postgraduate trainees: Experience of a teaching hospital. Pak Armed Forces Med J, 67(3), 439–445.** |
| --- |

Method: This prospective mixed methods study employed both quantitative and qualitative approaches to investigate the impact of Multi-Source Feedback (MSF). Data were collected using a modified mini-PAT Questionnaire comprising 12 closed-ended and 3 open-ended questions, supplemented by semi-structured interviews. The study involved two surveys administered three months apart to measure change. Quantitative data analysis utilized SPSS version 20, primarily employing the paired sample t-test to compare mean scores, with statistical significance set at p<0.05. Qualitative data from open-ended questions and interviews were analyzed using a content analysis technique to identify prevailing themes and patterns.

Sample: The study's participants were postgraduate trainees enrolled in FCPS/FRCS programs within the Department of Ophthalmology at Lahore General Hospital and Postgraduate Medical Institute, selected via non-probability convenience sampling, provided they met criteria regarding training duration. After exclusions, the final cohort consisted of 10 postgraduate trainees, predominantly in their third and fourth years of training. A diverse group of 24 raters from the same institution participated, including consultants, fellow postgraduate trainees, house officers, nursing staff, and paramedical personnel, offering a comprehensive 360-degree perspective on the trainees' attitudes.

Key findings: The study reported a statistically significant improvement in the overall attitude scores of postgraduate trainees following the implementation of MSF, with the mean score increasing from 4.00 ± 0.16 in Survey I to 4.43 ± 0.14 in Survey II (p=0.000). This overall enhancement suggests that MSF, when delivered effectively with attention to timeliness, confidentiality, and facilitative feedback, can positively influence trainee practice. While the ability to manage time effectively received the lowest scores, respecting patient's confidentiality consistently garnered the highest ratings across both surveys. Qualitative data further illuminated specific behavioral changes in some trainees in response to feedback regarding health habits and study time, although areas like punctuality and laziness showed varied responses, emphasizing the complex nature of attitudinal change facilitated by MSF.

| **2. Nathwani, J.N., Glarner, C. E., Law, K. E., McDonald, R. J., Zelenski, A. B., Greenberg, J. A., & Foley, E. F. (2017). Integrating Post-Operative Feedback into Workflow: Perceived Practices and Barriers. J Surg Educ, 74(3), 406–414.** [**https://doi.org/10.1016/j.jsurg.2016.11.001**](https://doi.org/10.1016/j.jsurg.2016.11.001)**.** |
| --- |

Method: This study utilized a cross-sectional survey design with a mixed methods approach, employing quantitative analysis of survey responses and qualitative coding of open-ended text responses to understand perceived practices and barriers regarding post-operative feedback. Surveys were developed using consensus agreement among a research team with prior survey experience and piloted before distribution. Quantitative data were analyzed using descriptive statistics, t-tests, and ANOVA, while qualitative text responses were analyzed using open coding to identify recurring themes, as described in Saldana (2005). The study took place over a one-month period, and data collection methods included an internet-based platform for residents and paper surveys for attendings, later coded into the same platform, with careful attention paid to maintaining format consistency.

Sample: The study sample consisted of general surgery residents (n=23, representing an 85% response rate out of 27 distributed surveys) and attending surgeons (n=22, representing a 92% response rate out of 24 distributed surveys) from a university-based general surgery department at a Midwestern academic medical center. Resident participants ranged from PGY 1 to PGY 5, with varying average case volumes per year and a majority female representation (61%). Attending surgeons had experience ranging from 1 to 30 years (mean 9.3 years), included various surgical sub-specialties within an academic practice, and were predominantly male (71% male, 29% female).

Key findings: The study revealed a significant discrepancy between the perceived current frequency of post-operative feedback and the desired frequency among both residents and staff surgeons, who largely shared similar opinions. Residents reported receiving feedback for procedure-specific performance after 25% of cases (staff reported giving after 34%), general technical feedback after 36% (staff after 32%), and non-technical performance after 17% (staff after 18%). In contrast, both groups desired procedure-specific and general technical feedback in over 80% of cases, and non-technical feedback in nearly 60% of cases. The preferred method and setting for feedback was overwhelmingly reported as verbal and immediately after the operation in the operating room. Key barriers identified by both groups included lack of time (71% of residents, 88% of staff), competing responsibilities at the end of the case (38% of residents, 73% of staff), the operating room environment being inappropriate for meaningful feedback (29% of residents, 53% of staff), and lack of interest from either party.

| **3. Bello, R. J., Sarmiento, S., Meyer, M. L., Rosson, G. D., Cooney, D. S., Lifchez, S. D., & Cooney, C. M. (2018). Understanding Surgical Resident and Fellow Perspectives on Their Operative Performance Feedback Needs: A Qualitative Study. J Surg Educ.** [**https://doi.org/10.1016/j.jsurg.2018.04.002**](https://doi.org/10.1016/j.jsurg.2018.04.002)**.** |
| --- |

Method: This qualitative study utilized a structured interview approach to explore surgical trainees' perspectives on operative performance feedback. Two trained research fellows conducted semistructured, one-on-one interviews with participants. Interview questions were initially drafted and iteratively updated based on emerging data, with prompts included to enhance comparability of responses. Detailed notes were taken during the interviews and maintained confidentially. Data analysis was thematic, with recurrent themes identified and iteratively updated. Both fellows independently coded themes from all interview notes, reached consensus through discussion, and organized findings into primary and secondary codes using a cut-and-paste technique from anonymized, aggregated data shared with the study team.

Sample: The study sample consisted of surgical residents and clinical fellows in general or plastic surgery across 9 academic training programs in the United States, representing all four census regions. Participants were recruited via email using purposive sampling to ensure representation across different levels of training (junior residents: PGY 1-3, senior residents: PGY 4-6, and clinical fellows). A total of 30 interviews were conducted, reaching theoretical saturation for the main topics of interest. The sample included 9 junior residents, 14 senior residents, and 7 clinical fellows, with 60% from plastic surgery and 40% from general surgery, and a gender distribution of 77% men and 23% women.

Key findings: The study found that operative performance feedback is considered very or extremely important by the vast majority (80%) of surgical trainees, with senior trainees more likely to rate it as extremely important. All trainees identified verbal, face-to-face feedback as the most valuable type, particularly when delivered during (92%) or immediately after (65%) surgical cases due to enhanced recall and relevance. While trainees acknowledged the utility of performance rating tools (used by 74% of residents), most (57% of users) viewed them positively primarily as a complement to, rather than a replacement for, verbal feedback and documentation, highlighting concerns about administrative burden and lack of nuanced scales. Trainees generally valued feedback more when received within one week of the operative event, perceiving delayed feedback (reported by 87%) as less valuable and potentially hindering improvement. Variability in feedback quality and timeliness was noted, attributed to attending surgeons' clinical comfort and available time.

| **4. Lees, M. C., Zheng, B., Daniels, L. M., & White, J. S. (2018). Factors Affecting the Development of Conﬁdence Among Surgical Trainees. J Surg Educ.** [**https://doi.org/10.1016/j.jsurg.2018.10.016**](https://doi.org/10.1016/j.jsurg.2018.10.016) |
| --- |

Method: This qualitative research study employed semi-structured interviews to explore factors influencing surgical residents' confidence from their perspective. Data collection involved a pre-interview "confidence relational map" activity to stimulate reflection, a single one-on-one semi-structured interview conducted by the lead researcher, and a post-interview member check for participant review of summaries. Interviews were audio-recorded, transcribed verbatim, and de-identified. Data analysis followed a thematic approach within a constructivist paradigm, involving iterative hand coding (descriptive and *in vivo*), categorization of codes in a spreadsheet, compilation of representative quotes, and development of themes from categories. A second coder independently reviewed transcripts, and the research group met multiple times to discuss results and develop a conceptual framework.

Sample: The study participants were volunteer residents from the University of Alberta General Surgery residency program in Edmonton, Alberta, Canada, specifically from postgraduate years (PGY) 2, 3, and 4. Residents in PGY-1, PGY-5, and those currently enrolled in dedicated research years were excluded due to potentially different experiences impacting confidence development. Out of 43 residents in the program, a total of 7 participated (3 PGY-2, 3 PGY-3, and 1 PGY-4), comprising 3 male and 4 female residents. Recruitment was voluntary, and the limited sample size from a single institution was acknowledged as a study limitation.

Key findings: The study identified that surgical confidence is shaped by a complex interplay of internal and external factors, influencing residents before, during, and after a surgical task. Key internal factors included personal experiences (especially operative), personal expectations (often leading to peer comparison), self-perception (particularly the fear of "not looking like an idiot" which hindered questioning), and individual skill development (recognizing personal improvement). Stress, mental health, and self-esteem played an indirect role via overall well-being, while personal life factors like marital status or children were not perceived as directly impacting surgical confidence. Important external factors encompassed patient characteristics (acuity, case difficulty, outcomes), receiving useful feedback (direct, timely, constructive), rapport with staff surgeons (feeling comfortable, trusted, and being allowed to problem-solve), and a supportive environment originating from all members of the surgical team, including senior back-up. Residents highlighted that negative experiences significantly undermined confidence, often feeling that confidence gains were slow and fragile ("20 steps back for each step forward"). The findings underscored the need for educators to focus on these identified factors, especially improving constructive feedback and fostering supportive environments, to enhance resident learning and accelerate their progression toward becoming confident, independent surgeons.

| **5. Dedhia, P. H., Barrett, M., Ives, G., Magas, C. P., Varban, O. A., Wong, S. L., & Sandhu, G. (2019). Intraoperative Feedback: A Video-Based Analysis of Faculty and Resident Perceptions. J Surg Educ, 76(4), 906–915.** [**https://doi.org/10.1016/j.jsurg.2019.02.003**](https://doi.org/10.1016/j.jsurg.2019.02.003)**.** |
| --- |

Method: This mixed methods study employed a video-based analysis coupled with validated surveys to investigate differences in resident and faculty perceptions of intraoperative feedback. Participants viewed a 10-minute video excerpt of a laparoscopic cholecystectomy and independently timestamped moments they perceived as feedback, subsequently completing surveys on the timing, amount, specificity, and satisfaction with operative feedback. Quantitative analysis included comparisons of overall timestamp counts (Mann-Whitney U test) and analysis of timestamp distribution in 10-second intervals (Student t tests), alongside analysis of survey responses (Student t tests), with statistical significance set at p<0.05. Qualitative data from free-text comments were also reviewed to characterize differently perceived moments.

Sample: The study involved 52 participants from the general surgery residency program at the University of Michigan (23 residents, 56% participation; 29 faculty, 88% participation). The resident cohort had a mean PGY level of 3.96 ± 2.2 years, with 30.4% being junior residents (PGY1-2). Participant demographics included 73.9% male residents and 65.5% male faculty. The video stimulus featured a laparoscopic cholecystectomy performed by a PGY2 resident and a faculty surgeon, chosen to provide ample opportunity for observable feedback events.

Key findings: The study confirmed that residents and faculty hold different perceptions of intraoperative feedback, particularly regarding its specificity and frequency, which residents perceived as lower compared to faculty in survey responses (p < 0.0005 for specificity). While the overall number of identified feedback moments during video review was not statistically different between groups, interval analysis revealed specific 10-second periods where faculty significantly more often identified interactions as feedback (p < 0.05). Notably, these faculty-identified moments frequently lacked explicit verbal labeling as "feedback" and often included nonverbal cues, suggesting that residents are less likely to interpret such implicit interactions as feedback or may perceive them as general teaching rather than personalized feedback, potentially leading to missed learning opportunities. Furthermore, free-text comments from residents indicated a preference for feedback delivery at the conclusion of the case, where they felt more receptive to digest and utilize the information for future improvement.

| **6. Gupta, A., Villegas, C. V., Watkins, A. C., Foglia, C., Rucinski, J., Winchell, R. J., Barie, P. S., & Narayan, M. (2020). General Surgery Residents’ Perception of Feedback: We Can Do Better. J Surg Educ, 77(3), 527–533.** [**https://doi.org/10.1016/j.jsurg.2019.12.009**](https://doi.org/10.1016/j.jsurg.2019.12.009)**.** |
| --- |

Method: This study employed a quantitative, cross-sectional design utilizing anonymous Likert scale surveys distributed electronically via REDCap to general surgery residents across university and community-affiliated hospitals within a single health system. The survey consisted of 25 questions assessing the frequency and perceived quality of feedback across the preoperative (PrO), intraoperative (IO), and postoperative (PO) periods. Data were tabulated and analyzed in Microsoft Excel using the Mann-Whitney U test, with statistical significance set at a = 0.05. Subanalyses were conducted to compare results between residents at university versus community hospitals, junior (PGY 1-3) versus senior (PGY 4-5) residents, and by gender.

Sample: The survey was distributed to 115 general surgery residents (GSRs), resulting in 83 completed responses (72% response rate), including both categorical and preliminary residents. The sample represented residents from a university hospital (n=49) and two community-affiliated hospitals (n=35). The distribution across training levels included PGY-1 through PGY-5 residents. The majority of respondents were categorical (86%) and male (58%). Participants were drawn from general surgery residency programs within NewYork Presbyterian-Weill Cornell Medical Center, NewYork Presbyterian-Queens, and NewYork Presbyterian-Brooklyn Methodist Hospital.

Key findings: The study revealed significant variability in the frequency and perception of feedback across the perioperative continuum, with a majority of residents reporting infrequent feedback, particularly outside the operating room (77% received PrO FB <= 20% of time, 24% IO FB <= 20%, and 64% PO FB <= 20%). Despite this, most residents found feedback helpful (58% found it helpful >= 80% of the time) and desired designated time for both PrO planning (82%) and PO debriefing (87%). An interesting finding was that residents at community hospital campuses reported receiving feedback more frequently across all perioperative phases compared to those at the university hospital (p < 0.05), suggesting potential cultural or structural differences in feedback delivery. Residents also perceived feedback from senior/chief residents as equally or more valuable than from attending surgeons, and a large majority noted that attending surgeons rarely explicitly stated when providing feedback (78% reported this occurred <= 20% of the time). Subanalyses by PGY level and gender showed no significant differences in feedback perception.

| **7. Vu, J. V., Harbaugh, C. M., De Roo, A. C., Biesterveld, B. E., Gauger, P. G., Dimick, J. B., & Sandhu, G. (2020). Leadership-Specific Feedback Practices in Surgical Residency: A Qualitative Study. J Surg Educ, 77(1), 45–53.** [**https://doi.org/10.1016/j.jsurg.2019.08.020**](https://doi.org/10.1016/j.jsurg.2019.08.020)**.** |
| --- |

Method: This exploratory qualitative study utilized semi-structured interviews with surgical residents. The interviews were audiotaped, transcribed, checked for accuracy, and de-identified. Data analysis involved manual line-by-line coding for both semantic and conceptual data by two independent researchers. Codes were finalized by consensus through discussion. Inductive reasoning was used to sort codes, identify patterns, and cluster them into themes and sub-themes. Member checking was performed with interview participants who were also research team members as a validity measure. NVivo 11 software was used for data analysis and management.

Sample: The study participants were 18 general surgery residents from a single residency program at the University of Michigan, a tertiary care academic institution. Residents were purposively selected to include key informants and ensure a balanced sample with respect to post-graduate year (PGY), gender, and race. The sample included residents from PGY 1 through PGY 7, representing 40% of the program. Eight participants identified as female (44%) and eleven as white (61%).

Key findings: The study identified four major themes regarding leadership-specific feedback in surgical residency. Firstly, residents strongly valued feedback for leadership development, recognizing its importance for improving day-to-day practice and overcoming "blind spots." Secondly, residents perceived current formal and informal feedback mechanisms as inadequate; formal feedback was often delayed, non-specific, and lacked focus on leadership skills, while informal feedback was infrequent, difficult to recognize, and subjectively interpreted. Thirdly, significant structural barriers (lack of opportunity, hierarchy) and personal/cultural barriers (reluctance to criticize, discomfort in giving/receiving feedback, taking feedback personally) hindered effective feedback exchange. Lastly, residents proposed recommendations including formalizing leadership-specific feedback from multiple sources, making it frequent, specific, and constructive, and utilizing mentors or coaching models to facilitate feedback reception and incorporation into action plans, thus fostering a more open feedback culture.

| **8. Neal, C. J., Durning, S. J., Dharmapurikar, R., McDaniel, K. E., Lad, S. P., & Haglund, M. M. (2022). From Their Eyes: What Constitutes Quality Formative Written Feedback for Neurosurgery Residents. J Surg Ed, 80(3), 323–330.** [**https://doi.org/10.1016/j.jsurg.2022.10.003**](https://doi.org/10.1016/j.jsurg.2022.10.003)**.** |
| --- |

Method: This study employed an embedded mixed methods design to explore neurosurgery residents' perceptions of written feedback, integrating quantitative and qualitative data. The quantitative analysis involved reviewing 2968 entries from the Surgical Autonomy Program (SAP) data from 2019-2021, analyzing the relationship between the use of the Zone of Proximal Development (ZPD) concept and residents' quality ratings of feedback using Chi-square tests. Qualitative analysis of the written feedback comments (2064 entries with comments) utilized a constant comparative technique with inductive coding performed by two independent researchers to identify thematic content. Quantitative data enhanced the qualitative findings by comparing feedback themes against resident-rated quality using Chi-square tests. Time-based data for evaluation completion was analyzed using the non-parametric Mann-Whitney U-test.

Sample: The study data originated from 2968 SAP entries collected between academic years 2019-2020 and 2020-2021 from two ACGME accredited neurosurgery training programs: Duke University (university-based) and the National Capital Consortium (NCC) (military). Duke contributed 2607 assessments (from 35 residents and 40 faculty) and NCC contributed 361 assessments (from 7 residents and 11 faculty). The dataset included assessments from residents across various PGY levels and case types, with individual resident identifiers removed to encourage candid feedback. Faculty and residents at both institutions received training on Vygotsky's theory and the SAP/TAGS scale implementation.

Key findings: The study identified five themes in written feedback: Non-Specific, Specific General Observations, Key Points, Next Steps, and Independent Practice. Feedback categorized as Specific General Observations, Key Points, or Independent Practice was significantly associated with higher perceived quality by residents compared to Non-Specific comments (p < 0.001). While Next Steps feedback trended towards higher quality than Non-Specific, it did not reach statistical significance (p = 0.17). Leaving the written comment section blank was associated with lower quality feedback than any specific feedback theme (p ≤ 0.001), although nearly 70% of blank evaluations were still rated valuable. The use of the ZPD concept was strongly associated with high quality feedback (91.4% when fully used vs 58.6% when not used, p < 0.001). Notably, comments indicating the attending did not remember the case performed significantly worse than even blank comments (44.4% valuable vs 69.8%, p = 0.01), highlighting the critical importance of the attending remembering the resident's performance. Feedback completion time was faster for blank or non-specific comments, and high-quality feedback was paradoxically associated with slightly faster completion times compared to low-quality feedback.

| **9. Rivard, S. J., Kemp, M. T., Evans, J., & Sandhu, G. (2022). Resident Perceptions of Faculty Behaviors Promoting Learner Operative Skills and Autonomy. J Surg Ed, 79(2), 431–440.** [**https://doi.org/10.1016/j.jsurg.2021.09.015**](https://doi.org/10.1016/j.jsurg.2021.09.015)**.** |
| --- |

Method: This qualitative retrospective analysis investigated resident-perceived faculty behaviors by utilizing anonymous open-ended comments from 623 department of surgery faculty teaching evaluations collected via MedHub and cross-referencing them with observed faculty entrustment scores from 196 OpTrust intraoperative observations of 28 faculty and 46 residents. The study employed a descriptive content analysis approach using NVivo12. Inductive open coding was performed by a single researcher (SJR), blinded to the faculty categories (promoting/limiting), to identify faculty behaviors. Codes were defined and refined iteratively. A consensus-building session with all research team members ensured agreement on resulting themes, which were then organized into categories based on similar behaviors. The relationship between these behavioral categories and the faculty performance categories was subsequently examined.

Sample: The study was conducted at a single tertiary midwestern allopathic academic medical center, analyzing data collected between January 2016 and August 2019. Participants included 46 resident surgeons (PGY 1-5) and 14 faculty surgeons across general, plastic, thoracic, and vascular surgery sections. The analysis specifically focused on comments from 355 faculty teaching evaluations and correlated them with performance attributes derived from 79 entrustment evaluations. Faculty were categorized as "promoting" (7 members) or "limiting" (7 members) based on quartiles of "percent push forward" (showing higher entrustment than resident entrustability) and "percent pull back" (showing lower entrustment than resident entrustability) scores derived from the OpTrust observations. Resident and faculty demographics were reported.

Key findings: The qualitative analysis highlighted that resident evaluations distinguished between faculty based on their personal traits, the working environment created, and their teaching techniques, categorizing them as "promoting" or "limiting" resident development. Promoting attendings were perceived as having positive personal traits (kind, funny, friendly, calm, patient), fostering a conducive learning environment (calm, relaxed, efficient, collaborative, respectful), and employing effective teaching techniques including preoperative goal setting, granting operative autonomy with "graduated responsibility" and allowing "productive struggle," and providing specific, actionable postoperative feedback. Conversely, limiting attendings were described with negative traits (condescending, disrespectful, unpredictable, impatient), created stressful and hostile operating room environments, and utilized ineffective techniques such as micromanaging, failing to delegate, treating residents as passive observers, overusing "show and tell," and providing unclear or sarcastic feedback. Interestingly, residents' perceptions of attendings' clinical knowledge, knowledge of literature, and technical skill were not found to be differentially associated with either the promoting or limiting categories. The study concludes that relationships, environment, and teaching techniques are paramount in resident development, advocating for faculty development focused on promoting behaviors.

| **10. Sisak, S., Salyer, C. E., Cortez, A. R., Vaysburg, D. M., Quillin III, R. C., & Van Haren, R. M. (2023). Experience of surgical subspecialty residents on general surgery rotations. The American Journal of Surgery, 225(3), 673–678.** [**https://doi.org/10.1016/j.amjsurg.2022.10.044**](https://doi.org/10.1016/j.amjsurg.2022.10.044) |
| --- |

Method: This study employed a retrospective analysis of ACGME operative case logs (2014-2021) for surgical subspecialty residents (urology, cardiothoracic, vascular, plastics) and categorical general surgery residents at a single institution, focusing on cases logged during core surgical rotations as first assistant or surgeon junior (PGY-1 and PGY-2 levels). Simulations, consults, and critical care experiences were excluded. Average case numbers per month on specific rotations were calculated by dividing the total cases by the total months spent on those rotations. A secondary component involved distributing separate surveys via RedCap to surgical subspecialty residents, program directors, and core surgery faculty to investigate perceived satisfaction, discrepancies in training, and overall quality of surgical education. Survey responses were voluntary, anonymous, and rated on a 5-point Likert scale. Statistical analysis utilized Wilcoxon rank sum tests for continuous data and descriptive statistics (total 'n' and percentage '%') for categorical data, with JMP Pro version 16.0. A p-value <0.05 was considered statistically significant. The study received Institutional Review Board approval.

Sample: The case log analysis included 50 residents: 29 general surgery residents (58%) and 21 subspecialty residents (42%). The demographic data for this group showed 23 females (46%) and 27 males (54%). The survey component had different response rates: 41% for the subspecialty resident survey (n=12 respondents, 92% male), 50% for the subspecialty program director survey (n=unknown number of respondents, 100% agreeing on certain points), and 27% for the core general surgery faculty survey (n=unknown number of respondents, all agreeing on certain points). The resident survey sample had a majority of respondents in Plastic Surgery (42%) and Cardiothoracic Surgery (33%), and a notable skew towards male (92%) and senior residents (PGY 3+).

Key findings: The study's primary finding from the case log analysis is that surgical subspecialty residents perform significantly fewer cases per month (median 13) on core general surgery rotations compared to categorical general surgery residents (median 21) (p < 0.001). This difference was particularly notable on acute care surgery, transplant surgery, and community general surgery rotations for R1 and R2 residents. Despite lower operative volume, the resident survey indicated that most subspecialty residents were satisfied with their off-service rotations (75%) and felt they were important for their training (58%). Key aspects valued by residents included working as a team member, having their input valued (67%), and receiving adequate faculty mentorship and beneficial feedback (83%). However, only half (50%) felt the total number of cases was adequate, and a majority (58%) felt the complexity was inadequate. Junior residents were less satisfied and felt their opinions were less valued than senior residents. Program directors universally agreed on the importance of these rotations but were split (50%) on the adequacy of case numbers/complexity and the need for more time. Core surgery faculty also agreed on the importance but largely felt the number/complexity of cases performed by subspecialty residents was inadequate (86%) and perceived that subspecialty residents showed less initiative to go to the operating room (71%). Potential reasons for the case volume discrepancy explored include less opportunity to scrub, more time on floor work, or less initiative from subspecialty residents, though faculty survey results did not strongly support the first two reasons. The study highlights that the value of core rotations extends beyond operative experience to include learning pre/post-operative care, managing complex patients, and developing teamwork skills. Receiving feedback was correlated with improved satisfaction in external literature cited.

| **11. Collings, A. T., Doster, D. L., Longtin, K., Choi, J., Torbeck, L., & Stefanidis, D. (2023). Surgical Resident Perspectives on the Preferred Qualities of Effective Intraoperative Teachers: A Qualitative Analysis. Acad Med, 98(5), 629–635.** [**https://doi.org/10.1097/ACM.0000000000005131**](https://doi.org/10.1097/ACM.0000000000005131)**.** |
| --- |

Method: This qualitative study utilized a grounded theory method with an inductive theoretical framework to explore surgical residents' perspectives on effective intraoperative faculty teaching qualities. Data were collected through 5 focus groups with categorical clinical general surgical residents of the same postgraduate year (PGY). Discussions were recorded, transcribed verbatim, and coded by a single author (ATC) who developed the codebook iteratively with subsequent coding. Codes were cleaned and consolidated collaboratively (ATC and DLD). Themes and subthemes were identified directly from the data, refined through virtual conferences with the entire working group, and supported by representative quotations. Data management was done using Microsoft OneNote and Excel. Reporting followed the Consolidated Criteria for Reporting Qualitative Research (COREQ) checklist.

Sample: The study participants were 39 categorical clinical general surgery residents from Indiana University School of Medicine, an urban academic medical center. They participated in 5 focus groups held from June to August 2021, representing an 80% overall participation rate across PGY levels 1 through 5. Participants per focus group ranged from 6 to 9 residents, with discussions lasting a median of 49 minutes. The sample included 19 female residents (48.7%). Focus group facilitators included a resident from another institution (ATC) and a resident from the same institution as participants (DLD), both with formal qualitative research training.

Key findings: The study identified six major themes and ten subthemes describing the ideal intraoperative teaching experience from the surgical residents' perspective. The six themes were: (1) Character (caring, respect for resident, self-control), (2) Intraoperative skill (clinical and operative skill, modeling leadership in the OR), (3) Instructional approach, (4) Feedback (content of feedback, debriefing), (5) Discernment of resident needs (managing expectations, individualizing instruction, autonomy), and (6) Variety of teachers. Residents particularly valued tangible strategies such as demonstrating genuine care, using clear directional language, and providing actionable feedback. They emphasized that an educator's character, including being able to discern their altruistic intentions, was crucial. Trust in the faculty's technical competence to ensure patient safety was foundational for a conducive learning environment. Effective instructional approaches involved specific and directional language, allowing for productive struggle, and demonstrating maneuvers. Feedback was most useful when actionable, distinguishing "good yelling" (constructive) from "bad yelling" (demeaning), and timely debriefing was highly valued, especially after complex cases. Residents appreciated faculty who could adapt their teaching to individual needs and set clear, consistent expectations, though autonomy was valued more when accompanied by explanation. A novel finding was the residents' appreciation for a diversity of teaching styles and personalities across different faculty members, recognizing that uncomfortable teaching can also be valuable for developing skills under pressure.

| **12. Go, M. R., Traugott, A. L., Ejaz, A., Collins, C., Harzman, A. E., Ellison, E. C., & Chen, X. (2024). Measuring Chief Resident Skill and Entrustment Progression in An Operative Coaching Program: Four Years’ Experience. J Surg Ed, 81(4), 457–464.** [**https://doi.org/10.1016/j.jsurg.2023.12.014**](https://doi.org/10.1016/j.jsurg.2023.12.014)**.** |
| --- |

Method: This mixed-methods study investigated the progression of operative skills and prospective entrustment (PE) in chief surgical residents within an operative coaching (OC) program. The quantitative portion retrospectively reviewed 441 validated performance evaluations (using procedure-specific Surgical Entrustable Professional Activities - SEPAs instruments) completed by residents, faculty coaches, and attending surgeons from July 2018 to June 2022. Key measures included Achieved Learning Goal (ALG), Procedural-Specific Skill (PSS), General Skill (GS), Step-Specific Guidance required (SSG - an autonomy measure), and PE, assessed using 5-point Likert scales. Pearson correlation coefficients and descriptive statistical analysis were used to assess longitudinal progression and relationships between variables, with p ≤ 0.05 considered statistically significant. The qualitative portion involved semi-structured interviews with graduated chief residents and faculty members, purposefully sampled for diversity, to understand their experiences and perceptions of OC and SEPAs. Interview data were recorded, transcribed, deidentified, and analyzed using a thematic framework method to reach thematic saturation. Written comments from SEPAs evaluations were also extracted for text analysis and incorporated to interpret findings.

Sample: The study data included 441 SEPAs evaluations from 147 OC cases involving 22 chief residents, 5 faculty coaches, and 24 attending surgeons. Of the chief residents, 50% were female, and the sample represented diverse ethnic backgrounds (54.5% Caucasian, 27.3% African American, 13.6% Asian, 4.5% Hispanic). The qualitative interviews included 5 graduated chief residents (3 female, varied ethnicity) and 5 faculty members (varied subspecialties). The OC cases included a variety of laparoscopic and open procedures (cholecystectomy, inguinal hernia, ventral hernia, partial colectomy).

Key findings: Quantitatively, the study found that resident GS (p = 0.036), SSG (p = 0.023), and PE (p = 0.002) significantly improved throughout the chief year in the OC program, while PSS did not show statistically significant improvement (p = 0.160). PE significantly correlated most strongly with SSG (r = 0.73, p < 0.0001) overall and in each quarter, followed by PSS (r = 0.59) and GS (r = 0.57). Chief residents on average underestimated their skills (PSS and GS) compared to attendings but perceived their PE at the same level as coaches and attendings, reaching a consensus of "Resident can lead the case" upon graduation. Attending surgeons tended to overestimate the autonomy they granted. Qualitatively, chief residents perceived OC as effective for improving self-regulated learning and particularly valued neutral authentic feedback, third-party real-time observation, and actionable feedback for practice readiness. Faculty noted OC promoted their engagement in skills assessment and teaching. The findings emphasize that autonomy (SSG) is a stronger predictor of PE than technical skills and that the OC program, utilizing SEPAs, facilitates resident learning, faculty teaching, and comprehensive assessment of skill, autonomy, and entrustment in the operating room.
