# Supplementary material for: Surgical Residents' Feedback Perceptions: A Scoping Review on Gaps and Improvements
Source: Clin Teach. 2025 Dec 15;23(1):e70323. doi: 10.1111/tct.70323 (PMC12706175; doi:10.1111/tct.70323)
Supplement: Supplementary file 3 — Appendix S3: Data extraction tool. [file TCT-23-e70323-s003.docx]

Appendix 3: Data extraction tool

***I. General Publication Information***

1. **Study ID:** (Sequential number or unique code for the study)
2. **Full Study Title:**
3. **Authors:** (List first author and corresponding author, if applicable, or main authors)
4. **Year of Publication:**
5. **Journal/Publication Source:** (Name of journal, publisher, etc.)
6. **Country of Origin of the Study:** (Where the research was conducted)
7. **Language of Publication:** (Confirm if English, Spanish, or Portuguese)
8. **Publication Type:** (Select one or more options)

( ) Primary Study (Qualitative, Quantitative, Mixed Methods)

( ) Systematic Review

( ) Meta-analysis

( ) Meta-synthesis

( ) Book/Book Chapter

( ) Guideline

( ) Other (Specify): ____________________________________________

1. **Inclusion Status:** (Mark if the study was included or excluded after full-text review)

( ) Included

( ) Excluded (Complete item 10)

1. **Reason for Exclusion (if applicable):** (e.g., does not meet population criteria, does not address feedback, not surgical context, language, publication type, etc.)

_______________________________________________________________

***II. Characteristics of the Studied Population***

1. **Total Number of Participants:** (Surgical Residents)
2. **Surgical Specialty(ies) of Residents:** (e.g., General Surgery, Orthopedics, Neurosurgery, etc.)
3. **Year(s) of Residency of Participants:** (e.g., PGY-1, PGY-2, PGY-3, all years, etc.)
4. **Age Range of Participants (if mentioned):** (e.g., mean age, range)
5. **Geographical/Institutional Context:** (e.g., university hospital, training center, country, region, etc.)

***III. Feedback Concept and Methodology***

1. **Definition of Feedback Used in the Study (if explicit):** (Transcribe or summarize the definition presented by the study authors)
2. **Type(s) of Feedback Investigated:** (Mark one or more options and elaborate)

( ) Oral

( ) Written

( ) Simulation-based

( ) Videotaping

( ) Audience Response Systems

( ) Computer-based Tools

( ) Patient Feedback

( ) Other (Specify): ___________________________________________________

1. **Feedback Delivery Methodology(ies) Addressed:** (Mark one or more options and elaborate)

( ) Feedback Sandwich

( ) Pendleton Model

( ) Pendleton Plus

( ) Learning Conversation

( ) Group Feedback

( ) Peer Feedback

( ) Multisource Feedback

( ) Self-feedback

( ) Other (Specify): ______________________________________________________

1. **Learning Domains Focused by Feedback (if mentioned):**

( ) Cognitive

( ) Psychomotor

( ) Affective

( ) Not specified

***IV. Residents' Perception of Feedback***

1. **Key Findings on Residents' Perception:** (Describe in detail the qualitative and quantitative results related to perception)
2. **Positive Aspects of Perception:** (e.g., feedback useful for improvement, generates action, valued, etc.)
3. **Negative Aspects of Perception:** (e.g., insufficient quantity, differing perception from giver, frustration, affects self-confidence, lack of specificity, etc.)
4. **Factors Influencing Perception (as per the study):** (Describe identified factors)

( ) Credibility of the Teacher/Giver

( ) Team Composition (multidisciplinary)

( ) Manner and Environment of Feedback Delivery

( ) Teacher's Educational Beliefs

( ) Learner's Individual Experiences

( ) Learner's Level of Expectation and Motivation

( ) Relationship between Feedback and Reflection (Internal Feedback)

( ) Other (Specify): _______________________________________________________

1. **Residents' Preferences Regarding Feedback (if mentioned):** (e.g., timing of delivery - **during/immediately after case; format - face-to-face; frequency - within 1 week)**
2. **How Perception was Measured in the Study:** (e.g., validated questionnaires, semi-structured interviews, focus groups, observation, Likert scales, etc.)

***V. Impact of Feedback on Residents' Education***

1. **Impact on Learning Progress:** (Describe how feedback affects residents' learning)
2. **Impact on Quality of Patient Care:** (If mentioned)
3. **Impact on Learner Engagement:** (If mentioned)
4. **Impact on Skill Development:** (e.g., surgical skills, communication, leadership, etc.)
5. **Impact on Self-confidence:** (If mentioned)
6. **Impact on Reflection/Internal Feedback:** (How external feedback stimulates self-assessment)

***VI. Study Methodology (of the article being extracted)***

1. Study Design: (e.g., Cross-sectional study, longitudinal, cohort, case-control, clinical trial, case study, action research, etc.)
2. Data Collection Methods: (e.g., Online questionnaires, in-person interviews, direct observation, document analysis, etc.)
3. Data Analysis Methods: (e.g., Descriptive statistical analysis, inferential; content analysis, thematic analysis, discourse analysis, etc.)
4. Main Tools/Instruments Used: (e.g., Specific scales, interview guides, qualitative/quantitative analysis software)

***VII. Knowledge Gaps and Implications***

1. **Knowledge Gaps Identified by the Study Authors:** (What the study suggests still needs to be investigated)
2. **Implications for Practice:** (Recommendations for educators, residency programs, curriculum development, etc.)
3. **Suggestions for Future Research:** (Directions that the study authors propose for future investigations)
