# Supplementary material for: Surgical Residents' Feedback Perceptions: A Scoping Review on Gaps and Improvements
Source: Clin Teach. 2025 Dec 15;23(1):e70323. doi: 10.1111/tct.70323 (PMC12706175; doi:10.1111/tct.70323)
Supplement: Supplementary file 4 — Appendix S4: Search strategies and results for all databases searched. [file TCT-23-e70323-s002.docx]

Appendix 4: Search strategies and results for all databases searched

| Database | Date and time of search | Number of articles found | Descriptors |
| --- | --- | --- | --- |
|  | March 15, 2024 |  |  |
| Medline | 15:02h | 149 | (“Formative Feedback” OR “Feedback, Formative” OR “Constructive Feedback” OR “Feedback, Constructive” OR “Feedback (Learning)”) AND (“Medical Staff, Hospital” OR “Hospital Medical Staff” OR “Hospital Medical Staffs” OR “Staff, Hospital Medical” OR “Staffs, Hospital Medical” OR “Medical Staffs, Hospital” OR “Physicians, Junior” OR “Junior Physician” OR “Junior Physicians” OR “Physician, Junior” OR “Registrars, Hospital” OR “Hospital Registrar” OR “Hospital Registrars” OR “Registrar, Hospital” OR “Attendant Physicians, Hospital” OR “Hospital Attendant Physician” OR “Hospital Attendant Physicians” OR “Attendant Physician, Hospital”) AND (“Teaching” OR “Training Techniques” OR “Training Technique” OR “Technique, Training” OR “Techniques, Training” OR “Training Technics” OR “Technic, Training” OR “Technics, Training” OR “Training Technic” OR “Pedagogy” OR “Pedagogies” OR “Teaching Methods” OR “Teaching Method” OR “Method, Teaching” OR “Methods, Teaching” OR “Academic Training” OR “Training, Academic” OR “Training Activities” OR “Training Activity” OR “Activities, Training” OR “Activity, Training” OR “Techniques, Educational” OR “Educational Techniques” OR “Educational Technique” OR “Technique, Educational” OR “Educational Technics” OR “Educational Technic” OR “Technic, Educational” OR “Technics, Educational”) AND (“General Surgery” OR “Surgery, General” OR “Surgery”) AND (“Perception”) |
| DOAJ^a^ | 15:08h | 167 |  |
| ROAD^b^ | 15:15h | 118 |  |
| ASP^c^ | 15:20h | 63 |  |
| BMC^d^ | 16:18h | 31 |  |
| WILEY^e^ | 16:21h | 60 |  |

^a^Directory of Open Access Journals

^b^Directory of Open Access Scholarly Resources

^c^Academic Search Premier

^d^BioMed Central Open Access

^e^Wiley-Blackwell
