# Supplementary material for: Surgical Residents' Feedback Perceptions: A Scoping Review on Gaps and Improvements
Source: Clin Teach. 2025 Dec 15;23(1):e70323. doi: 10.1111/tct.70323 (PMC12706175; doi:10.1111/tct.70323)
Supplement: Supplementary file 5 — Appendix S5: Tables summarising the results. [file TCT-23-e70323-s005.docx]

Appendix 5: Tables summarizing the results

Table 1. General publication Information

| Study ID | Year of publication | Authors | Full study title | Journal/publication source | Country of origin of the study | Publication type | Study design | Study methodology |
| --- | --- | --- | --- | --- | --- | --- | --- | --- |
| 01/2017 | 2017 | Zahid Kamal; Uzma Iqbal; Suhair Akhlaq; Amna Adil; Muhammad Ramzan | Does Use  of Multi-Source Feedback (MSF) Affect the Attitudes of Postgraduate Trainees: Experience of a Teaching Hospital | Pakistan Armed Forces Medical Journal | Pakistan | Primary study | Prospective mixed method study (quantitative & qualitative) | The study used a modified mini-peer assessment tool (mini-PAT) questionnaire, available in English or Urdu (for paramedicals), which consisted of 12 close-ended questions and 3 open-ended questions. It also included semi-structured interviews where needed. Data collection was conducted in two surveys: Survey I, followed by Survey II, which was conducted three months after the first to determine any change. Qualitative data from interviews were analyzed by identifying themes and patterns using content analysis technique. For quantitative analysis, Statistical Package for the Social Sciences (SPSS®) version 20 was used, applying descriptive statistics (frequencies and percentages) and paired sample t-test to compare residents’ mean scores. |
| 02/2017 | 2017 | J.N. Nathwani; C. E. Glarner; Katherine E. Law; R. J. McDonald; A. B. Zelenski; J. A. Greenberg; E. F. Foley | Integrating Post-Operative Feedback into Workflow: Perceived Practices and Barriers | Journal of Surgical Education | United States of America (USA) | Primary study | This study employed a cross-sectional survey design to investigate perceptions regarding post-operative feedback. It utilized a mixed methods approach, analyzing both quantitative survey data and qualitative text responses from open-ended questions. | Data were collected through surveys administered to general surgery residents and attending surgeons. Residents received surveys via an internet-based platform (Qualtrics®), while attendings received paper surveys that were later coded into Qualtrics®. This method was chosen based on historical response rates at the institution. The surveys inquired about the current and desired frequency of post-operative feedback, preferred methods, and perceived barriers. Text responses from open-ended questions were also collected. |
| 01/2018 | 2018 | Ricardo J. Bello; Samuel Sarmiento; Meredith L. Meyer; Gedge D. Rosson; Damon S. Cooney; Scott D. Lifchez; Carisa M. Cooney | Understanding Surgical Resident and Fellow Perspectives on Their Operative Performance Feedback Needs: A Qualitative Study. | Journal of Surgical Education | USA | Primary study | Qualitative research study employing semistructured, one-on-one interviews. The researchers utilized an iterative process for thematic analysis as data emerged from interviews. | Semistructured, one-on-one interviews were conducted with surgical trainees. These interviews took place either in person or over the phone and lasted 20 to 30 minutes. Interviewers took notes during the discussions, and these notes were analyzed thematically. |
| 02/2018 | 2018 | Mackenzie C. Lees, Bin Zheng, Lia M. Daniels, and Jonathan S. White | Factors Affecting the Development of Confidence Among Surgical Trainees. | Journal of Surgical Education | Canada | Primary study | Qualitative research study using semi-structured interviews. The data analysis employed thematic analysis within a constructivist paradigm. | The study utilized semi-structured, one-on-one interviews with surgical residents. Data collection also involved a pre-interview activity (confidence relational map) and a post-interview research brief/member check. The interviews were audio-recorded, transcribed verbatim, and deidentified. The analysis method was thematic analysis, involving coding, grouping into categories, and developing themes. A second coder was used for review. |
| 01/2019 | 2019 | Priya H. Dedhia; Meredith Barrett; Graham Ives; Christopher P. Magas; Oliver A. Varban; Sandra L. Wong; Gurjit Sandhu | Intraoperative Feedback: a Video-Based Analysis of Faculty and Resident Perceptions. | Journal of Surgical Education | USA | Primary study | Mixed-methods study (quantitative and qualitative). The article employs video-based analysis combined with surveys (validated survey and open-ended questions). Participants timestamped moments of perceived feedback while watching a video and completed a survey on feedback perceptions. Data from timestamps and surveys were analyzed quantitatively and qualitatively. The study design involved comparing perceptions between residents and faculty. | Participants (residents and faculty) independently reviewed a 10-minute video segment of a laparoscopic cholecystectomy and timestamped instances they identified as feedback using iMovie®. They then completed an online modified survey with Likert scale and open-ended questions regarding their perioperative feedback experiences and the feedback observed in the video. Video timestamp data was exported and analyzed quantitatively, including a 10-second interval analysis. Survey responses were collected electronically and analyzed quantitatively using Student t tests (comparing resident vs. faculty perceptions). Free text comments from the survey were reviewed for content (qualitative analysis) to support quantitative findings. |
| 01/2020 | 2020 | Aakanksha Gupta; Cassandra V. Villegas; Anthony C. Watkins; Christopher Foglia; James Rucinski; Robert J. Winchell; Philip S. Barie; Mayur Narayan. | General Surgery Residents’ Perception of Feedback: We Can Do Better. | Journal of Surgical Education | USA | Primary study | Quantitative, cross-sectional survey study. Anonymous surveys using Likert scales were distributed to general surgery residents to assess the frequency and perceived quality of feedback across the preoperative (PrO), intraoperative (IO), and postoperative (PO) periods. Comparisons were made between university and community hospital settings, junior and senior residents, and by gender. | Anonymous electronic surveys consisting of 24 Likert scale questions about feedback frequency and perceived usefulness, and 1 question about the usefulness of designated discussion time, were distributed via Research Electronic Data Capture (REDCap®). Data were tabulated using REDCap® and analyzed in Microsoft Excel™ using the Mann-Whitney U test, with a statistical significance level set at alpha = 0.05. Subanalyses comparing different groups (university vs. community, junior vs. senior, male vs. female) were conducted. Completion of the survey was voluntary and anonymous. |
| 02/2020 | 2020 | Joceline V. Vu; Calista M. Harbaugh; Ana C. De Roo; Ben E. Biesterveld; Paul G. Gauger; Justin B. Dimick; Gurjit Sandhu | Leadership-Specific Feedback Practices in Surgical Residency: a Qualitative Study | Journal of Surgical Education | USA | Primary study | Exploratory qualitative study utilizing semi-structured interviews. The study performed a secondary coding analysis of interview transcripts, focusing on the intersection of leadership and feedback. Thematic analysis was used to identify major themes. | Semi-structured, one-on-one interviews were conducted with surgical residents at a single institution. The interviews were audiotaped, transcribed verbatim, and deidentified. A secondary coding analysis was performed on these transcripts, specifically focusing on leadership and feedback, using line-by-line coding by two independent researchers. Codes were finalized by consensus, and inductive reasoning was used for thematic analysis, clustering codes into themes and sub-themes. Member checking was performed as a validity measure. NVivo 11® software was used for data analysis and management. |
| 01/2022 | 2022 | Chris J. Neal; Steven J. Durning; Rajeev Dharmapurikar; Katherine E. McDaniel; Shivanand P. Lad; Michael M. Haglund | From Their Eyes: What Constitutes Quality Formative Written Feedback for Neurosurgery Residents | Journal of Surgical Education | USA | Primary study | Embedded Mixed Methods Design. The study primarily used a qualitative approach (constant comparative qualitative methodology with textual thematic analysis of written feedback) and embedded quantitative analysis (Chi-square tests, Mann-Whitney U-test) to enhance the qualitative findings by examining relationships between feedback themes, zone of proximal development (ZPD) use, and resident-rated feedback quality. | Data from the Surgical Autonomy Program (SAP) online assessment tool (academic years 2019-2021) were reviewed. The SAP tool involves attendings providing written feedback and rating residents on the TAGS (Teach and demonstrate, Advise and scaffold, Guide and monitor, Solo and observe) scale after operative cases. Residents, upon receiving the written feedback, categorize its quality (significant, valuable, limited, none) and report attending's ZPD use (full, partial, none). The written feedback text was analyzed qualitatively using a constant comparative method and inductive coding to identify themes (non-Specific, specific general observation, key points, next steps, independent practice). Quantitative analysis then examined the relationship between these themes, resident-rated quality (categorized as valuable/significant vs. limited/none), and the extent of ZPD use, using statistical tests. Time taken for evaluation completion was also analyzed. |
| 02/2022 | 2022 | Samantha J Rivard; Michael T. Kemp; Julie Evans; Gurjit Sandhu | Resident Perceptions of Faculty Behaviors Promoting Learner Operative Skills and Autonomy | Journal of Surgical Education | USA | Primary study | Qualitative retrospective analysis. The study utilized anonymous open-ended comments from surgical residents on faculty teaching evaluations and cross-referenced them with observed faculty entrustment scores obtained using the OpTrust® tool. A descriptive content analysis approach was applied to the resident comments. | The study involved a retrospective analysis of two data sources: anonymous open-ended comments from residents on 623 department of surgery faculty teaching evaluations (collected via MedHub®, Jan 2016-Aug 2019) and observed faculty entrustment scores from 79 OpTrust® IO observations (Jan 2016-Aug 2019) for a subset of these faculty. Faculty were categorized as "promoting" or "limiting" based on OpTrust® scores (percent pull back/push forward). Resident comments from 355 evaluations related to these faculty were imported into NVivo12®. Inductive open coding was performed on the comments by one researcher, blinded to faculty categories. Codes were reviewed and finalized by consensus with the research team. Codes were organized into categories and themes, which were then assessed within the context of promoting/limiting faculty categories. This method aimed to correlate subjective resident evaluations with objective IO observations. |
| 01/2023 | 2023 | Stephanie Sisak; Christen E. Salyer; Alexander R. Cortez; Dennis M. Vaysburg; R. Cutler Quillin III; Robert M. Van Haren | Experience of Surgical Subspecialty Residents on General Surgery Rotations | The American Journal of Surgery | USA | Primary study | Mixed-methods (retrospective case log analysis and cross-sectional survey). The article utilizes both quantitative analysis of case logs and survey data (Likert scale and categorical data) and qualitative aspects via interpretation of survey responses. | Operative case logs from the Accreditation Council for Graduate Medical Education (ACGME) database for general surgery residents and surgical subspecialty residents (urology, cardiothoracic, vascular, plastics) from 2014-2021 were retrospectively analyzed, cross-referenced with rotation schedules to identify cases logged as first assistant and surgeon junior on core general surgery rotations. Simulations, consults, and critical care were excluded. Average cases per month were calculated and compared using Wilcoxon rank sum tests. Separate surveys were distributed via RedCap® to subspecialty residents (11 statements), program directors (7 statements), and core surgery faculty (8 statements), using a 5-point Likert scale to assess their perceptions of these rotations, including aspects related to feedback. Responses were voluntary and anonymous. Categorical and continuous data from the surveys were reported and analyzed statistically (Wilcoxon rank sum tests) with significance at p < 0.05. |
| 02/2023 | 2023 | Amelia T. Collings; Dominique L. Doster; Krista Longtin; Jennifer Choi; Laura Torbeck; Dimitrios Stefanidis | Surgical Resident Perspectives on the Preferred Qualities of Effective Intraoperative Teachers: a Qualitative Analysis | Academic Medicine | USA | Primary study | Qualitative analysis using grounded theory method. | The study utilized focus groups with categorical clinical general surgery residents to explore their perspectives on ideal IO faculty teaching behaviors and qualities. Five focus groups were conducted and divided by postgraduate year (PGY). Discussions were audio-recorded using Zoom® and transcribed verbatim. Data analysis followed grounded theory principles with an inductive theoretical framework. Transcriptions were coded line-by-line by a single author, with a codebook developed iteratively. Codes were cleaned and consolidated collaboratively. Themes and subthemes were identified directly from the data, refined through virtual conferences, and supported by representative quotations. Reporting followed consolidated criteria for reporting qualitative research (COREQ) guidelines. |
| 01/2024 | 2024 | Michael R. Go; Amber L. Traugott; Aslam Ejaz; Courtney Collins; Alan E. Harzman; E. Christopher Ellison; Xiaodong (Phoenix) Chen | Measuring Chief Resident Skill and Entrustment Progression in an Operative Coaching Program: Four Years’ Experience | Journal of Surgical Education | USA | Primary study | Mixed-methods study. The quantitative part of the study examined the longitudinal progression and correlation of resident operative skill (procedural-specific and general), autonomy (step-specific guidance required - SSG), and prospective entrustment (PE) using data from surgical entrustable professional activities (SEPAs) evaluations. The qualitative part used semi-structured interviews with participants (graduated chiefs and attendings) and text analysis of written comments from evaluations to understand their experiences and perceptions of the operative coaching (OC) program and SEPAs instruments. | Quantitative data from 441 SEPAs evaluations across 147 OC cases from July 2018 to June 2022 were extracted. SEPAs measures included achieved learning goal (ALG), procedural-specific skill (PSS), general skill (GS), SSG (autonomy measure), and PE. Statistical analysis involved calculating Pearson correlation coefficients to determine relationships between variables and PE, and descriptive statistical analysis (using JMP Pro®) to investigate progression over the chief year quarters. Qualitative data included semi-structured interviews with 5 graduated chief residents and 5 attendings (recorded, transcribed, deidentified) and 357 written comments from SEPAs evaluations. Thematic analysis (using a framework method) was performed on interviews and text analysis was done on written comments. Quantitative and qualitative results were combined for interpretation. |

Table 2. Characteristics of the studied population

| Study ID | Sample size | Resident’s specialty | Year(s) of residency of participants | Age range of participants | Geographical/institutional context |
| --- | --- | --- | --- | --- | --- |
| 01/2017 | The study initially enrolled 15 postgraduate trainees (PGRs), but final results were based on data from 10 PGRs, after excluding 3 who moved to other hospitals and 2 with inadequate responses. Participants were FCPS (fellowship of the college of physicians and surgeons) trainees. | Ophthalmology | Of the 10 PGRs included in the final results, eight were in year 3 and two were in year 4 of their four-year training period. | Not mentioned | The study was conducted in the Department of Ophthalmology at Lahore General Hospital and Postgraduate Medical Institute in Lahore, Pakistan. |
| 02/2017 | Twenty-three general surgery residents participated in the study. | General surgery | Resident participants ranged from PGY 1 to PGY 5. | Not mentioned | The study was conducted within a general surgery department at a 566-bed academic medical center located in the Midwestern United States. The context involves residents rotating through a tertiary care center, a children's hospital, and a veteran's hospital, supervised by full-time general surgery staff. |
| 01/2018 | Thirty participants. Specifically, this included 9 junior residents, 14 senior residents, and 7 clinical fellows. | General surgery and plastic surgery | Participants included junior residents (PGY 1-3), senior residents (PGY 4-6), and clinical fellows. | Not mentioned | The study involved surgical residents and clinical fellows from departments or divisions of general or plastic surgery at USA academic institutions. Participants were affiliated with Johns Hopkins University School of Medicine (17 participants) and 8 other academic training programs (13 participants) across the 4 census regions of the USA. |
| 02/2018 | Seven participants | General surgery | PGY 2, 3, and 4. Residents in PGY-1, PGY-5, and those in dedicated research years were excluded. | Not mentioned | University of Alberta Hospital, a tertiary care center in Edmonton, Alberta, Canada. |
| 01/2019 | Fifty-two participants. This included 23 residents (56% participation rate) and 29 faculty members (88% participation rate). | General surgery. | PGY 1-5. The average PGY level for residents was 3.96. The distribution was: PGY 1 (17.4%), PGY 2 (13.0%), PGY 3 (13.0%), ADT (academic development time) year 1 (13.0%), ADT year 2 (13.0%), PGY 4 (13.0%), PGY 5 (17.4%). ADT counts towards residency training years. Junior residents (PGY 1-2) represented 30.4% of respondents. | Not mentioned | The study was primarily conducted at the University of Michigan Hospital and Health Systems in Ann Arbor, Michigan. Faculty participants also included those from Dartmouth-Hitchcock in Lebanon, New Hampshire, but data collection (video viewing and surveys) took place at the University of Michigan. It involved participants from academic surgery departments. |
| 01/2020 | Eighty-three respondents out of 115 general surgery residents surveyed. This represents a 72% response rate. | General surgery residents. This included both categorical and preliminary residents. | PGY 1 through 5. Residents were categorized as junior (PGY 1-3) and senior (PGY 4-5) for comparison. The breakdown of the 83 respondents by year was: 22 PGY-1s, 19 PGY-2s, 10 PGY-3s, 13 PGY-4s, and 11 PGY-5s. | Not mentioned | The study was conducted within a single, urban integrated health system in New York, involving surgical residency programs at: New York Presbyterian-Weill Cornell Medical Center (a university hospital), New York Presbyterian-Queens (a university-affiliated community hospital), New York Presbyterian-Brooklyn Methodist Hospital (a university-affiliated community hospital) Participants were from these 3 institutions. 49 residents were from the university hospital, and 35 from the 2 community hospitals. |
| 02/2020 | Eighteen participants | General surgery | PGY 1 through 7. The study aimed for a balanced sample with respect to PGY, and the results indicate representation from PGY 1, 2, 3, 4, 5, 6, and 7. | Not mentioned | The study was conducted within a single general surgery residency program at the University of Michigan, a tertiary care, academic institution in Ann Arbor, Michigan. |
| 01/2022 | 2968 SAP entries (evaluations). These entries were from 42 unique residents (7 at National Capital Consortium - NCC, 35 at Duke) and 51 unique faculty (11 at NCC, 40 at Duke) over academic years 2019-2021. | Neurosurgery | The study included data from residents across all PGY levels. The analysis included "All SAP assessments during this time frame... regardless of case type, PGY level, and institution". While PGY level was mentioned in the context of assessing resident progress along the TAGS scale, the *sample* for this study (the 2968 entries) was not stratified or analyzed by PGY level. | Not mentioned | The study was conducted at two academic institutions in the United States: Duke University, Durham, North Carolina (a university-based residency program) and NCC (the military's neurosurgery residency program). Affiliations listed include Walter Reed National Military Medical Center in Bethesda, Maryland. |
| 02/2022 | Data were analyzed from 355 faculty teaching evaluations containing resident comments and linked to 79 OpTrust® entrustment evaluations. These evaluations were associated with 46 surgical residents and 14 faculty surgeons whose OpTrust® scores allowed categorization into "promoting" or "limiting" groups. | General surgery, plastic surgery, thoracic surgery, and vascular surgery | PGY 1 through 5. The sample included residents from all PGY levels. | Not mentioned | The study was conducted at a single tertiary midwestern allopathic academic medical center in the United States, which is the University of Michigan. |
| 01/2023 | 50 residents were included in the case log analysis (29 general surgery, 21 subspecialty). Surveys were distributed, with a response rate of 41% for subspecialty residents (n=12), 50% for program directors (n=4, based on 8 statements and assuming responses cover the 7 statements asked), and 27% for core surgery faculty (n=8 based on 8 statements). | General surgery (for comparison in case log analysis) and Surgical Subspecialties (urology, cardiothoracic, vascular, and plastics) as the primary population of interest for both case logs and surveys. | Case log data for junior residents (R1 and R2) was analyzed as most subspecialty residents did not rotate off-service as mid-level or senior residents. The resident survey included junior residents (PGY-1 and PGY-2) and senior residents (PGY-3 or higher). | Not mentioned | The study was conducted at a single institution, the University of Cincinnati, Department of Surgery, in Cincinnati, Ohio, USA. This is an academic institution. |
| 02/2023 | Thirty-nine participants | General surgery | Participants were from all clinical PGY: PGY-1, PGY-2, PGY-3, PGY-4, and PGY-5. Focus groups were conducted with residents from a single PGY class at a time. | Not mentioned | The study was conducted at Indiana University School of Medicine, an urban academic medical center. The focus groups took place at this institution. One of the interviewers was from the University of Louisville, another institution. |
| 01/2024 | Quantitative: 441 SEPAs evaluations from 147 OC cases completed by 22 chief residents, 5 faculty coaches, and 24 attending surgeons. Qualitative: 5 graduated chief residents and 5 faculty members were interviewed. | General surgery | Chief resident year (typically PGY-5). The study specifically focused on chief residents in their final year of training. | Not mentioned | The study took place at The Ohio State University Wexner Medical Center General Surgery residency program in Columbus, Ohio, USA. This is a university-based academic medical center. |

Table 3. Feedback concept and methodology

| Study ID | Definition of feedback used in the study (if explicit) | Feedback method | Feedback delivery methodology(ies) addressed | Learning domains focused by feedback (if mentioned) |
| --- | --- | --- | --- | --- |
| 01/2017 | Not mentioned | MSF or 360˚ feedback. | Raters received a training session on how to respond to the proforma items. If there were any ambiguities, the rater was invited for a discussion to clarify those grey areas. Participating registrars also received a training session explaining the MSF process. After Survey I, face-to-face feedback sessions were conducted, where participants were debriefed by one of the authors. The confidentiality of the raters was strictly maintained. | The feedback primarily focused on attitude components such as receptivity, response, and internalization of a situation. It also addressed communication and interpersonal skills, resource utilization, reliability, professionalism, and response to adverse situations. The questionnaire questions included: awareness of own limitations, awareness to respond to psychosocial aspects of patient care, appropriate utilization of resources, ability to manage time effectively, communication with patients and their families, respecting patient's confidentiality, communication with colleagues, ability to recognize contribution of others, accessibility/reliability, and attitude in adverse circumstances. |
| 02/2017 | Not mentioned | Not mentioned | Not mentioned | The feedback types investigated specifically address technical skills (procedure-specific and general technical) and non-technical skills (communication, teamwork, leadership). The introduction also frames these within the context of the ACGME core competencies. |
| 01/2018 | Not mentioned | Not mentioned | Not mentioned | The feedback discussed by trainees focuses on several learning domains within surgical training: 1) Operative performance and technical skills; 2) Clinical judgment and clinical decision-making; 3) Identifying and addressing deficiencies in performance; 4) Skill acquisition and surgical competence; 5) Quality of care provided to patients (mentioned in the introduction and by a junior resident). The paper also links feedback to ACGME competencies: practice-based learning and improvement, interpersonal and communication skills, and patient care. |
| 02/2018 | Not mentioned | Not mentioned | Informal (verbal) and formal (written) | The study implies that feedback in surgical training focuses on: 1) Operative performance and technical skills: Residents discussed feedback in the context of performing surgeries and identifying what they were doing wrong technically and how to fix it; 2) Clinical judgment and decision-making: Although less explicitly detailed than technical skills, the context of surgical tasks implies feedback would cover these areas; 3) Identifying and correcting errors/deficiencies: Feedback is discussed as a way to know what you are doing wrong and how to improve. |
| 01/2019 | Not mentioned | Not mentioned | Verbal and nonverbal interactions | The study specifically mentions feedback related to: 1) Technical skills: The context is laparoscopic surgery and the free text comments also refer to technical aspects and specific actions; 2) Knowledge of anatomy: This was explicitly a subject area of feedback queried in the survey, and there was a statistically significant difference in perception frequency between residents and faculty regarding feedback on knowledge of anatomy; 3) Clinical judgment/decision-making: While not a primary focus, the introduction mentions guided discovery learning which incorporates interactive feedback into practice patterns (related to learning and judgment). One resident commented about getting little feedback focused on "less-important aspects of the procedure," implying they desire feedback on more critical aspects. |
| 01/2020 | Not mentioned | Not mentioned | Not mentioned | The feedback discussed pertains to several learning domains relevant to surgical training: 1) Perioperative care: The overall focus of the feedback; 2) Operative performance/Technical skills: IO feedback is directly related to operative skills. PO feedback time can be used to discuss "operative skills, including ergonomics, instrument handling, respect for tissue, and procedural knowledge and flow"; 3) Judgment and Decision-Making: PO designated time is also seen as crucial to review judgment and decision-making; 4) Identifying areas for improvement: Feedback serves to guide and improve future performance. The paper also lists ACGME competencies under "COMPETENCIES," implying feedback relates to these areas: patient care, medical knowledge, practice-based learning and improvement, interpersonal and communication skills, professionalism, systems-based practice. |
| 02/2020 | The introduction refers to a definition of feedback from existing literature: "information about a learner’s performance intended to improve performance". While this definition is cited, the study itself focuses on residents' *perceptions* of what constitutes and is effective leadership-specific feedback rather than explicitly using this definition as a framework provided to participants. | Not mentioned | The study examines resident perceptions of various feedback delivery methods for leadership: 1) Formal Methods: Predominantly monthly end-of-rotation evaluations and semi-annual review of written comments. Residents perceive these as suboptimal due to being delayed, nonspecific, and not focused on leadership; 2) Informal Methods: Includes in-person, on-the-fly feedback (perceived as rare but valuable when it occurs), and subjective, subtle processes like reading team members' facial expressions, body language, or paying attention to team dynamics and patient outcomes (perceived as difficult to recognize and interpret). | The primary learning domain is leadership development, specifically leadership performance and skills (emotional intelligence, team-building, conflict management). The study contrasts this with clinical and technical skills, noting that leadership is often not the focus of formal feedback. The ACGME competencies of interpersonal and communication skills and professionalism are also mentioned in relation to leadership requirements. |
| 01/2022 | The study does not explicitly state a single definition of feedback used *within* its design or provided to participants. However, it operates within the context of existing literature definitions, citing Ende (1983) on principles of good feedback (teacher/trainee as allies, timely, expected, based on first-hand data, remediable behaviors, descriptive non-evaluative language) and referring to van de Ridder *et al.*'s definition in the Discussion ("information on a trainee’s observed performance in comparison with a standard in order to improve the trainee’s performance"). The study focuses on residents' *perception* of the *quality* of the written feedback they received. | Not mentioned | The study addresses feedback delivery in the context of operative cases via the SAP tool: 1) Form: Primarily focuses on written feedback. Verbal feedback during the case is mentioned as context but was not analyzed. Nonverbal feedback (e.g., pointing) was identified as a theme in the written comments by researchers, highlighting its inclusion in the content of the feedback analyzed, even if not the method of delivery studied directly; 2) Tool/Context: The feedback is delivered within the structure of the SAP tool, which is based on Vygotsky's ZPD and utilizes the TAGS scale. Faculty underwent training on using the SAP and providing written feedback. | The feedback examined in this study focuses on aspects of surgical performance and development, aligning with key learning domains: 1) Operative Performance/Technical Ability: The SAP tool and TAGS scale assess operative performance and progressive independence in completing a procedure. Themes like "Specific General Observation," "Key Points" about procedures, and "Independent Practice" relate directly to technical and procedural skills; 2) Progress Towards Independence: The ZPD concept and TAGS scale are centered on advancing the resident's ability to perform independently. Feedback themes like "Independent Practice" and "Next Steps" directly address this; 3) Identifying Deficiencies/Areas for Improvement: Good feedback, in the context of ZPD, helps identify what the resident needs to do to advance. Themes like "Next Steps" explicitly focus on areas to work on; 4) Knowledge: Feedback can relate to learning points about the procedure, indicating a focus on medical or procedural knowledge. The ACGME competency of Practice-Based Learning and Improvement is listed, which is supported by effective feedback. |
| 02/2022 | The study does not provide a formal, explicit definition of "feedback" used in the study design or provided to residents. However, it implicitly addresses the concept by examining resident comments about faculty teaching behaviors, specifically focusing on "PO feedback" as a teaching technique. The Discussion section cites other literature's definition of feedback as "information on a trainee’s observed performance in comparison with a standard in order to improve the trainee’s performance." The study's analysis of comments characterizes what residents perceive as effective or ineffective feedback within the context of operative training. | Not mentioned | Verbal feedback is described, including direct, practical, and granular feedback. The study analyzes written comments from evaluations which describe these interactions. Implicit feedback is also captured through descriptions of attendings' personal traits and the environment created. | Operative Skills and Autonomy: This is the central focus of the study. Feedback directly impacts the development of operative skills and the progression of autonomy/entrustment. |
| 01/2023 | Not mentioned | Not mentioned | Not mentioned | Not mentioned |
| 02/2023 | Not mentioned | Not mentioned | Not mentioned | The feedback discussed by residents primarily focuses on: 1) Technical Skills: IO feedback is perceived as better for technical skills, including specific actions, adjusting hands, and suturing skills; 2) Clinical Judgment/Decision-Making: PO feedback is seen as better for understanding "how am I doing" and solidifying understanding about cases and decisions made. Constructive criticism, even if harsh, was tolerated if the purpose was to improve technical skills or clinical judgment; 3) Identifying and Addressing Deficiencies: Feedback helps residents know what they should be doing and how to perform techniques correctly. Debriefing helps solidify understanding and fill in gaps. |
| 01/2024 | Not mentioned | Not mentioned | The study addresses feedback delivery in the context of the OC program: 1) Provider: Primarily from a faculty coach, but also potentially from the attending surgeon via the shared SEPAs evaluation report and comments; 2) Form: Verbal and/or written feedback. Qualitative results highlight coaches' written feedback having more diverse terms than attending surgeons'; 3) Source Material: SEPAs evaluations and aggregated reports serve as the basis for the directed feedback. 4) Observation Context: The coach observes the operative case in "real time without intervening," which informs the feedback provided later. | The feedback within the OC program and SEPAs framework focuses on several learning domains relevant to surgical practice: 1) Operative Skills: Assessed via PSS and GS measures; 2) Autonomy: Assessed via SSG required; 3) Entrustment: Assessed via PE; 4) ALG: Feedback relates to how well a resident achieved their case-specific learning goal; 5) Specific Operative Aspects: Residents valued feedback on specific points like "how I was holding the laparoscopic equipment, how I set up the room, my body language, the height of the table, etc." and observed details like posture and tendency to look for instruments; 6) Self-Regulated Learning: Residents described OC as effective in improving self-regulated learning, implying feedback contributes to this metacognitive skill; 7) Ergonomics and Emotional Intelligence: Residents appreciated feedback on aspects "not often covered," including these areas. |

Table 4. Residents' perception of feedback

| Study ID | Key findings on residents' perception | Positive aspects of perception | Negative aspects of perception | Factors influencing perception (as per the study) | How perception was measured in the study |
| --- | --- | --- | --- | --- | --- |
| 01/2017 | There was an overall improvement in residents' attitudes, indicating that MSF leads to a change in practice, especially when delivered accurately, timely, with confidentiality maintained, and facilitative feedback. The mean difference in overall score between the two surveys was statistically significant (p-value=0.000), showing an overall improvement in scores from Survey I to Survey II. Concerns raised in Survey I (such as smoking habits and being overweight) were addressed by participants in Survey II, with reduced smoking and initiation of physical exercise. Similarly, issues like time spent on studies and punctuality showed some improvement after feedback. | Most residents accepted feedback amicably, and there was a positive correlation between self-scoring and other raters' scoring. Positive traits identified in candidates included good manners (friendly, soft-spoken, down to earth, less talkative, helpful), personality (honest, sincere, generous, trustworthy, dependable, smiley face, hardworking, pleasant, witty), and skills (good information technology – IT - skills, research, good at studies, efficient time management). | One resident denied smoking when confronted with feedback, and another reacted aggressively, wanting to know the identity of the rater who gave corrective feedback. The study noted that failure to ensure secrecy can trigger rivalry or hatred among co-workers. | Perception and acceptance of feedback are influenced by the accurate and timely delivery of MSF, maintenance of confidentiality, and the presence of facilitative feedback sessions. The credibility of feedback sources and the alignment of feedback with the individual's self-perception of performance are also important factors. | Perception was measured by comparing residents' mean scores on a modified mini-PAT questionnaire (12 close-ended questions) before (Survey I) and after (Survey II, three months later) the feedback intervention. Additionally, 3 open-ended questions and semi-structured interviews were used to collect qualitative data on traits and concerns, which were analyzed to identify themes and patterns. |
| 02/2017 | General surgery residents perceive post-operative feedback as highly valuable and believe it should be delivered significantly more frequently than it currently is. They report receiving procedure-specific, general technical, and non-technical feedback less often than they desire. Residents prefer verbal feedback immediately after the operation in the operation room (OR) as the best method, but identify several barriers to this occurring, primarily time, competing responsibilities, and lack of privacy. | Residents perceive post-operative feedback as having "high educational value" and "highly value" it. They express a strong desire for increased frequency of feedback, perceiving that both procedure-specific and general technical feedback should occur after over 80% of cases, and non-technical feedback after nearly 60% of cases. Residents also identify verbal feedback immediately post-operation in the OR as the "best practice" or "most ideal method and setting". | Residents perceive a significant discrepancy between the current frequency of receiving feedback and the desired frequency. They report receiving procedure-specific feedback after only 25% of cases, general technical feedback after 36%, and non-technical feedback after 17%. They also identify substantial barriers that prevent feedback from occurring as often as desired or in the ideal setting. These barriers include time, competing end-of-case responsibilities, the operating room being an inappropriate environment (lack of privacy), and perceived lack of interest. | The study highlights several factors that residents perceive as influencing the delivery and frequency of feedback, which in turn shapes their perception. These are primarily the identified barriers: 1) Time: perceived lack of time or need to move quickly post-operation; 2) Competing Responsibilities: different tasks residents and attendings must complete at the end of a case; 3) Inappropriate Environment: the presence of other personnel in the OR making it difficult to give/receive meaningful or critical feedback; 4) Lack of Interest: perceived lack of interest from the attending surgeon. | Resident perception was measured quantitatively and qualitatively through a survey instrument. The survey asked residents for estimated percentages regarding how often they *have received* and *think they should receive* different types of post-operative feedback (procedure-specific, general technical, non-technical). It also included an open-ended question about perceived barriers and a ranking question for ideal feedback methods/settings. |
| 01/2018 | Key findings reveal that surgical trainees consider operative performance feedback very or extremely important. They overwhelmingly prefer verbal, face-to-face feedback, ideally delivered during or immediately after a surgical case. While valuing timeliness (within 1 week), they recognize unavoidable delays. Performance rating tools are seen as potentially useful for formative feedback and documentation but should serve as a complement rather than a replacement for direct verbal interaction. Trainees also perceive significant variability in the quality, timeliness, and credibility of feedback provided by attendings. | Trainees hold several positive views regarding feedback: 1) Importance: Most perceive feedback as very or extremely important for their training (80%); 2) Preferred Type: Verbal, face-to-face feedback is considered the most valuable due to ease of understanding and ability to cover technical aspects. Written feedback is valued for documentation; 3) Preferred Timing: Feedback during (92%) or immediately after (65%) cases is strongly preferred as it's fresh and allows for better communication; 4) Value of Delayed Feedback (within limits): Feedback is generally still useful within 1 week or even as end-of-rotation reviews; 5) Performance Rating Tools: Many view these tools positively, finding them useful for objective measurement, comparing performance against peers (especially junior residents), serving as a "back-up log," providing "objective" feedback, and potentially prompting immediate face-to-face feedback. | Trainees also expressed negative perceptions and concerns regarding feedback: 1) Delayed Feedback: A large majority (87%) reported feedback delays sometimes. Delayed feedback is considered less valuable (71%), potentially irrelevant, not taken seriously, interfering with learning and teaching, and capable of prolonging bad skills. One junior resident felt feedback became useless after a day if they couldn't remember the event; 2) Performance Rating Tools: Concerns include increased administrative burden ("one more thing," "an extra step"), perception that scores are too simplistic and don't catch nuances of different training levels and actual skills, lack of objectively anchored scales, and lack of user-friendliness. There is also a strong view that these tools should *not replace* verbal feedback; 3) Variability: Trainees perceive variability in the quality, timeliness, and credibility of feedback depending on the attending's ability, comfort, and available time. | Several factors influence how residents perceive feedback: 1) Type of Feedback: Verbal, face-to-face feedback is perceived as more valuable than written or tool-based feedback when used in isolation; 2) Timing of Delivery: Immediate feedback (during or right after a case) is perceived as more valuable, accurate, objective, and actionable than delayed feedback. Timeliness within a week is still seen as useful; 3) Quality of Feedback: "Good feedback" is perceived as objective, current, evaluative, formative, accurate, and not easily dismissed as mere opinion; 4) Attending Characteristics: The attending's perceived clinical comfort, available time, ability to teach, and commitment to providing feedback significantly influence the trainee's perception of feedback quality and timeliness; 5) Level of Training: Junior, senior residents, and fellows may have slightly different perspectives on the importance and focus of feedback (e.g., junior residents emphasizing behavioral change, senior residents focusing on deficiencies, fellows on clinical decision-making); 6) Performance Rating Tool Design: The user-friendliness of the tool, whether it uses objectively anchored scales, and its ability to capture the nuances of different skill levels influence trainees' views on its usefulness. | The perception of residents and fellows was measured using a qualitative research approach. This involved conducting semistructured, one-on-one interviews with surgical trainees. The interview questions were designed to explore trainees' perspectives on feedback type, timing, value, and their views on performance rating tools. Data analysis was thematic, identifying recurring themes from the interview notes to understand trainee views. Quantitative proportions were used to summarize how many participants held certain views (e.g., percentage reporting feedback as very important). |
| 02/2018 | Residents perceive feedback as a highly important external factor influencing their confidence. They value feedback that is direct, useful, regular, and constructive, particularly when it explains *why* something was wrong and *how* to fix it. Positive feedback boosts confidence significantly. However, residents noted that feedback quality varies among attendings and that receiving negative feedback without guidance on improvement can be detrimental to confidence. The perception of the staff-resident relationship also heavily influences how feedback is received and impacts confidence. | Residents perceive the following aspects of feedback positively: 1) Usefulness and Importance: Feedback is seen as highly important for their development and crucial for knowing how to improve; 2) Positive Reinforcement: Positive feedback is a significant confidence booster; 3) Constructive Guidance: Negative feedback is valued if it is delivered constructively and includes explanation and guidance on correction; 4) Regularity and Directness: Feedback that is regular and direct is perceived as more useful. | Residents perceive the following aspects of feedback negatively: 1) Lack of Constructive Guidance: Receiving negative feedback that points out errors but does not explain *why* or *how to fix it* is perceived negatively and not helpful; 2) Impact on Confidence: Poorly delivered feedback (e.g., critical without being constructive) can lower confidence; 3) Variability in Quality: Feedback quality varies among attendings, which can be frustrating; 4) Relationship with Staff: A poor relationship with staff can amplify the negative impact of perceived errors and feedback, further lowering confidence. | Several factors influence how residents perceive feedback: 1) Content and Constructiveness: Feedback that explains *why* and *how* to improve is perceived much more positively than feedback that only points out errors; 2) Formality and Regularity: While both informal and formal feedback are mentioned, the study emphasizes the need for direct, useful, and regular feedback; 3) Source: Feedback comes from staff surgeons, senior colleagues, and peers. The relationship and rapport with the staff surgeon is particularly highlighted as influencing the perception of feedback and overall confidence; 4) Timing (post-task): Feedback received after task completion appears to be perceived as most important for confidence development. | Resident perception was measured through qualitative data collected via semi-structured, one-on-one interviews. The interview guide included questions specifically designed to explore experiences with confidence during surgical training, which involved discussions about factors influencing confidence, including feedback. Residents' responses during these interviews, as transcribed and analyzed thematically, formed the basis for understanding their perceptions of feedback and its role in confidence development. |
| 01/2019 | Surgical residents perceive IO feedback differently than faculty. Specifically, residents perceive feedback as less frequent, less specific, and occurring with less immediacy compared to faculty. Using video review, the study found that residents identify fewer IO interactions as feedback than faculty, particularly interactions that are nonverbal or not explicitly labeled as feedback. Residents may interpret these interactions more as general IO teaching rather than personalized feedback. Free text comments also suggest residents may be more receptive to receiving feedback at the conclusion of the case rather than during it. Despite these differences in perceived quantity/quality, there was no significant difference in overall satisfaction with operative feedback between residents and faculty in this study. | Residents in this study showed no statistically significant difference in *overall satisfaction* with operative feedback compared to faculty. This suggests that despite perceiving lower frequency, specificity, and immediacy, they might still be relatively satisfied, perhaps valuing the feedback they *do* receive or prioritizing effective interactions over quantity. | Residents perceived operative feedback as occurring with significantly decreased immediacy, specificity, and frequency compared to faculty. They identified fewer feedback events during the video review, particularly in specific 10-second intervals where faculty identified significantly more. Residents were less likely to interpret nonverbal interactions or interactions not explicitly labeled as feedback as actual feedback, sometimes viewing these more as general teaching. One resident felt they received little feedback, often focusing on less important aspects. This disparity in identification is seen as potentially limiting effective IO learning. | Key factors influencing how residents perceive IO interactions as feedback include: 1) Position: Simply being a resident versus a faculty member influences how interactions are observed and experienced; 2) Explicitness/Labeling: Interactions that are not overtly labeled as "feedback" are less likely to be identified as such by residents; 3) Nonverbal Cues: Residents are less likely than faculty to identify nonverbal interactions (like pointing) as feedback; 4) Interpretation (Feedback vs. Teaching): Residents may interpret interactions as general IO *teaching* rather than personalized *feedback*, especially if they are not explicitly linked to the trainee's goals; 5) Timing Preference: Residents' suggestion that they may be more receptive to feedback postoperatively implies that the timing of delivery influences their perception and receptiveness during the procedure itself. | Perception was measured using a combination of methods: 1) Video Timestamping: Participants reviewed a surgical video and marked the specific moments (via timestamps) they perceived as feedback, providing a quantitative measure of the frequency and timing of perceived feedback events; 2) Survey (Likert Scale): A modified, previously validated survey was administered asking participants to rate their perceptions of current perioperative feedback experiences regarding aspects like timing, amount, specificity, and satisfaction using a 5-point Likert scale; 3) Survey (Free Text Comments): Open-ended questions in the survey allowed participants to provide qualitative comments explaining their perceptions and experiences with feedback. The combination of these methods allowed for both quantitative comparison of perceived frequency/characteristics and qualitative exploration of the *reasons* behind differing perceptions. |
| 01/2020 | General Surgery Residents perceive feedback as important and generally helpful, but its frequency is perceived as low, particularly outside the OR. A significant portion of residents feel feedback is lacking in all perioperative phases. Residents strongly desire designated, structured time for both PrO planning and PO debriefing. They perceive feedback from senior residents as more constructive and valuable than from attending surgeons. Attending surgeons are perceived as rarely explicitly stating feedback or creating a safe environment for questions. Residents at community hospitals report receiving feedback more frequently than those at university hospitals. | Residents perceive feedback as generally helpful, with a majority finding it useful most of the time (58% >= 80% of the time). They see feedback from senior/chief residents as particularly valuable and constructive. Residents express a strong desire for dedicated, structured time for feedback sessions both before and after cases (82% PrO, 87% PO), highlighting a positive view of planned feedback opportunities. They also view feedback in all perioperative phases as important for successful outcomes and future cases. | Residents perceive the overall frequency of feedback received as low (46% <= 20% of the time). They feel feedback is particularly lacking intraoperatively and in all three perioperative areas (52%). They perceive that attending surgeons rarely explicitly state when giving feedback (79% <= 20%) or rarely create a safe environment for questions (63% <= 20%). While valuing senior resident feedback, they perceive senior residents as seldom guiding them through cases (63% <= 20%). There is a perceived disparity between the amount of feedback faculty report giving and what residents feel they receive and recognize. Some residents prefer natural conversation (51%), but a large portion still prefer explicit statements (44%), indicating that current methods are not universally meeting preferences. Written feedback is rarely preferred (2%). | Factors influencing residents' perception of feedback include: 1) Timing/Perioperative Phase: Perception of frequency varies significantly based on whether feedback is PrO, IO, or PO. Residents' desire for designated time also highlights the importance of timing; 2) Institution Type: Residents at community hospitals perceive receiving feedback more frequently across perioperative phases than those at university hospitals. The authors suggest a potential difference in "culture of learning" may play a role; 3) Feedback Provider: The source of feedback (attending vs. senior resident) influences perceived constructiveness and value. Senior residents are perceived more positively in this regard. Rapport with senior residents is suggested as a factor; 4) Explicitness: Attending surgeons explicitly stating they are providing feedback influences resident recognition. Lack of explicit labeling may lead residents to not perceive interaction as feedback; 5) Learning Environment: The presence of a "safe environment to ask questions" influences interactions. The "learning environment" theory emphasizes psychological comfort for maximum learning and trust; 6) Format/Structure: Residents' preference for structured formats and designated times indicates these influence perceived effectiveness and desirability; 7) Culture: A broader "feedback culture" or lack thereof is seen as an overarching factor influencing the provision and reception of feedback. Differences in "culture of learning" between settings are also proposed; 8) Faculty Factors (Potential): Although not assessed, faculty comfort, experience, and preparedness with providing feedback are suggested as potential influences on variability and resident perception. Attending attitude towards teaching is also linked to resident evaluations of OR teaching. | Resident perception was measured using a cross-sectional survey design with anonymous electronic questionnaires distributed via REDCap®. The survey included Likert scale questions asking residents to rate their experiences regarding the *frequency* and *perceived usefulness/quality* of feedback received in different perioperative phases (PrO, IO, PO) and from different sources (attending vs. senior residents). It also assessed their views on the importance of feedback, the perceived presence of a safe environment for questions, and their preferences for feedback format and timing. The collected quantitative data from the Likert scales were analyzed statistically (Mann-Whitney U test) to compare perceptions between groups. |
| 02/2020 | Surgical residents strongly value leadership-specific feedback for their development, recognizing it helps identify blind spots and calibrate self-perceptions. However, they perceive current formal and informal feedback mechanisms for leadership as inadequate—formal feedback is often delayed, nonspecific, and doesn't focus on leadership, while informal feedback is difficult to recognize and interpret. Significant structural (lack of opportunity, hierarchy) and personal/cultural barriers (discomfort giving/receiving, taking comments personally, culture of avoiding offense) hinder effective feedback exchange. Residents desire a more formalized, frequent, specific system for leadership feedback from multiple sources, preferably reviewed with a mentor, and believe this would foster a more open feedback culture. | Residents perceive leadership feedback as very important for their development and day-to-day practice improvement. They understand the need to actively listen to and incorporate feedback. In-person, on-the-fly feedback, though rare, is perceived as valuable when received because it is timely. Residents strongly desire better feedback mechanisms, indicating a positive view of the *potential* and *value* of effective leadership feedback. | Residents perceive formal leadership feedback as suboptimal because it is: 1) Delayed (losing usefulness for timely behavior change); 2) Nonspecific ("Good job!" is not helpful); 3) Not focused on leadership skills, despite leadership being an expected competency; 4) Often from faculty who don't observe their leadership directly. Informal feedback is perceived as difficult to recognize and interpret, and highly subjective. Personal and cultural barriers are seen negatively as they prevent honest, constructive feedback. Taking feedback personally is a negative experience, amplified by the stressors of residency. | Several factors influence residents' perception of leadership-specific feedback: 1) Source: Formal evaluations (from faculty/peers) vs. informal cues (peers, attendings, team members' non-verbals). Perception of formal sources not observing leadership; 2) Timing: Delayed feedback is perceived as less useful than timely, in-person feedback; 3) Specificity: Nonspecific feedback is perceived as unhelpful; 4) Focus: Feedback not explicitly focused on leadership skills is perceived as inadequate for leadership development; 5) Delivery Method: Verbal, in-person feedback is perceived as valuable. Written feedback is seen as delayed. Subtle informal cues are difficult to recognize/interpret; 6) Relationship/Rapport: A trusted relationship (e.g., with a mentor) is perceived as facilitating the reception and incorporation of feedback; 6) Environment/Culture: The hierarchical nature of surgery and a culture that discourages forthright, critical feedback negatively influence the process. A culture built around regular feedback is perceived as potentially mitigating defensiveness; 7) Personal Factors (Receiver): The resident's ability to be open to feedback, separate leadership skills from personality, and handle criticism influences how feedback is received; 8) Personal Factors (Giver): The comfort level and ability of faculty/peers to give specific, constructive feedback influences what is delivered and how it is perceived. | Resident perception was measured through a qualitative research approach using semi-structured, one-on-one interviews. Although the initial interviews were part of a larger study on leadership development, feedback emerged as a recurring topic. A secondary coding analysis was then performed specifically on the interview transcripts, focusing on residents' statements about leadership and feedback. Thematic analysis was used to identify and characterize residents' perceptions, experiences, and suggestions related to leadership-specific feedback. |
| 01/2022 | Neurosurgery residents perceive written feedback quality based significantly on its content and the faculty's approach. They rate feedback as higher quality when it contains specific observations about their performance, highlights key learning points, or indicates progress towards independence. Non-specific comments or leaving the written section blank result in lower perceived quality, though leaving it blank was sometimes still rated Valuable/Significant (69.8%). Critically, faculty comments indicating they *did not remember* the resident's performance were perceived very negatively, even worse than blank feedback. Utilizing the ZPD concept when providing feedback also correlates with higher perceived quality. Residents found timely feedback to be better, with quicker faculty completion times associated with higher perceived quality, though the median time was 7.1 hours. | Residents perceive feedback positively when it is specific to their performance, highlights key learning points, or acknowledges their readiness for independent practice. The use of a theory-based approach like ZPD by faculty is perceived very positively, significantly increasing the likelihood of high-quality feedback perception. Even simply providing *any* written comment (beyond blank) is seen more positively than leaving it blank (except for "next steps" theme, which was similar to non-specific). Receiving feedback relatively soon after the case is also perceived positively. | Residents perceive feedback negatively or as having less value when it is non-specific or the written comment section is left blank. Comments explicitly stating the attending did not remember the resident's performance are perceived particularly poorly, receiving the lowest rate of "valuable to significant" ratings. Feedback focusing *solely* on "next steps" was not perceived as significantly better than non-specific feedback. Longer faculty completion time for the assessment is associated with lower perceived feedback quality. The study also notes that variability in feedback quality exists. | The perception of feedback quality is influenced by: 1) Content Specificity: Written comments detailing specific performance, key points, or independence are key predictors of high-quality perception; 2) Absence of Content: Blank written sections or non-specific comments lead to lower perceived quality compared to specific content; 3) Explicit Lack of Recall: Comments like "I don't remember the details" are highly detrimental to perceived quality; 4) Faculty's Use of Theory (ZPD): When faculty report utilizing the ZPD concept during the case, residents are significantly more likely to perceive the feedback as high quality; 5) Timeliness of Faculty Completion: While the overall median time was several hours, quicker completion of the online evaluation by faculty is associated with higher perceived quality; 6) Social Contract/Relationship: A breakdown in the "social contract" (e.g., faculty not remembering the resident's performance) negatively impacts perception. The importance of the teacher and trainee working as allies is highlighted; 7) Focus (ZPD Alignment): Feedback focusing *solely* on steps beyond the resident's current ZPD might be perceived as less valuable; 8) Completeness of Written Feedback: Contrary to intuition, having multiple feedback themes within one written comment did not significantly improve perceived quality compared to a single specific theme. | Resident perception of written feedback quality was measured through the residents' own categorization within the SAP system. Upon receiving the online written feedback from the attending, residents were prompted to rate the quality of that feedback by selecting one of four predefined categories: "significant feedback both stretching/affirming," "some feedback but valuable," "some feedback but limited," and "no significant feedback." This resident-assigned rating served as the primary measure of their perception. Additionally, residents reported whether the attending had utilized the ZPD concept (fully, partially, none), which was also a measure of their perception regarding the underlying educational approach. |
| 02/2022 | Residents' perception of faculty's effectiveness in promoting their development, including through feedback, is strongly influenced by the attending's personal traits, the learning environment created, and the teaching techniques employed, rather than solely their clinical/technical expertise. Residents perceive faculty who exhibit positive traits (kind, patient, calm), create supportive environments (calm, teamwork, respectful), and use effective teaching techniques (goal setting, postop debrief, constructive feedback, appropriate autonomy/productive struggle) as promoting their development. Conversely, those with negative traits (condescending, unpredictable), creating stressful environments (hostile, tense), and using ineffective techniques (micromanaging, observer role, unclear expectations, sarcastic feedback) are perceived as limiting. Residents highly value constructive feedback that is specific and actionable. | Residents positively perceive faculty who are "kind," "funny," "friendly," "calm," "patient," "level-headed," and "down to earth." They value environments that are "calm," "relaxed," and foster "teamwork and collaboration" where they feel "comfortable" and treated with "respect." They appreciate teaching techniques that include PrO goal setting, structured PO debriefs, and constructive feedback that is "direct," "practical," "granular," "detailed," and "specific," allowing them the chance to enact it. They also value appropriate operative autonomy and the opportunity for "productive struggle." | Residents negatively perceive faculty who are "condescending," "disrespectful," "unnecessarily impatient," "angry," "easily frustrated," "unpredictable," "difficult to read," or have "poor communication skills." They dislike environments that are "hostile," "tense," "stressful," "menacing," or involve "mistreatment" of the team. Negative teaching techniques include "micromanaging," "lack of delegation," "inability to give up control," treating residents as "observers" or reducing them to passive roles, overutilizing the "show and tell" method, and having "unclear expectations" or providing "sarcastic" or indirect feedback. These behaviors lead to strained relationships and make it difficult to learn. | Resident perception of faculty's impact on their development, including through feedback, is primarily influenced by: 1) Attending's Personal Traits: Kindness, patience, calmness, sense of humor, approachability; 2) Learning Environment: Whether it is perceived as calm, relaxed, supportive, respectful, and promotes teamwork versus being tense, stressful, or hostile; 3) Teaching Techniques: Effectiveness and constructiveness of methods like goal setting, debriefing, feedback delivery (directness, specificity, actionability), delegation, and granting appropriate autonomy; 4) Communication Skills: Clarity of expectations, ability to explain reasoning, and overall communication style; 5) Relationship/Rapport: The quality of the relationship between the resident and attending significantly impacts how feedback is received and the overall learning experience; 6) Attending's Focus: Whether the attending focuses on resident development or primarily on control/efficiency. | Resident perception was measured retrospectively through qualitative analysis of anonymous open-ended comments written by residents on faculty teaching evaluations. These free-text comments, submitted through a standard medical education management system (MedHub®) after surgical service rotations, captured residents' subjective views and experiences with faculty behaviors, including feedback. The researchers categorized faculty based on objective entrustment scores and then analyzed the comments related to those categorized faculty, using descriptive content analysis to identify themes reflecting resident perceptions. |
| 01/2023 | Subspecialty residents perceive receiving appropriate feedback from general surgery attendings as beneficial to their learning, with a large majority (83%) agreeing or strongly agreeing with this statement. This perceived benefit correlates with overall satisfaction with their off-service rotations. | The primary positive aspect is the perception that receiving "appropriate feedback" from general surgery attendings is "beneficial to their learning," reported by 83% of subspecialty resident survey respondents. This perceived benefit is linked to higher overall satisfaction with the rotations. | Not mentioned | The study highlights that receiving *any* feedback (specifically, "appropriate feedback") from general surgery attendings is a factor influencing residents' perception that the feedback is beneficial to their learning (83% agreement). The study also correlates receiving feedback with overall satisfaction with the rotation, suggesting that the presence of feedback positively influences their overall experience perception. The feedback provider (general surgery attendings) is an implicit factor. | Resident perception regarding feedback was measured using a cross-sectional survey. Subspecialty residents were asked to respond to a statement using a 5-point Likert scale (ranging from strongly disagree to strongly agree) indicating whether they agreed that they received appropriate feedback from general surgery attendings that was beneficial to their learning. This quantitative measure (percentage agreeing/disagreeing) captured their perception. |
| 02/2023 | Surgical residents perceive effective IO teaching, including feedback, as dependent on both the technical expertise and character of the attending, as well as their instructional approach and ability to discern resident needs. Regarding feedback specifically, residents highly value it when it is actionable, constructive (even if critical), timely (IO for technical, PO for overall), and helps them understand *why* they are doing something wrong and *how* to fix it. They distinguish constructive criticism ("good yelling") from demeaning comments ("bad yelling"). Debriefing after challenging cases is particularly valued. They acknowledge that effective teaching styles, including feedback delivery, vary among attendings and that diversity in teaching approaches is beneficial. | Residents perceive the following aspects of feedback and teaching positively: 1) Actionability: Feedback is most critical when it is actionable, telling them exactly what to do and giving them a chance to do it; 2) Constructiveness: Constructive criticism, even if perceived as harsh or "yelling," is valued if the *intent* is to improve skills and judgment. Feedback that explains *why* and *how* to fix something is highly appreciated; 3) Timeliness (Appropriate): IO feedback is valued for technical skills, and timely PO debriefing is valued for overall performance and challenging cases; 4) Debriefing: Structured time, even after long cases, to discuss decisions and outcomes is highly valued for consolidating learning and setting future goals; 5) Affirmative Feedback: Positive reinforcement highlighting correct technique is appreciated; 6) Variety of Teachers: Exposure to diverse teaching styles and methods is seen as a strength of residency, leading to becoming a well-rounded surgeon. They value learning different ways to perform procedures; 7) Teacher's Character/Environment: Attending displaying caring, respect, self-control, clinical skill, and modeling leadership creates a conducive learning environment where residents feel safer and more comfortable focusing on learning. | Residents perceive the following aspects of feedback and teaching negatively: 1) Demeaning/Personal Attacks: "Bad yelling" that involves demeaning comments or personal attacks is not helpful and diminishes opportunity for improvement; 2) Lack of Constructiveness: Feedback that only points out what's wrong without explaining *why* or *how to fix it* is not helpful. Attending taking over without any constructive feedback is particularly awful; 3) Vague/Nonspecific Language: Using non-anatomical terms or vague goals like "provide more tension" is not effective. Specific, directional language is preferred; 4) Lack of Caring/Respect: Feeling like they are being "used" as an assistant rather than being genuinely taught or respected is a negative perception of the teacher's character. Lack of respect from the attending leads to lack of respect from the team; 5) Lack of Self-Control/Unpredictability: Attendings who lose self-control, get pissed off, or have a history of volatility increase resident stress and decrease ability to learn; 6) Lack of Trust in Attending's Skill: Feeling that the attending does not have a clear plan or technical expertise to fix errors is prohibitive to learning and creates anxiety; 7) Taking Over Without Explanation: Attendings taking over a case without explaining *why* they are doing so or what the resident could have done differently is seen as awful and assumes the resident should learn just by watching. | Resident perception of effective IO teaching and feedback is influenced by: 1) Teacher's Character: Attending's perceived caring, respect for the resident, and self-control. Residents can intuit the intent behind critical feedback; 2) Teacher's IO Skill: Attending's clinical and operative skill and ability to model leadership. Residents need to trust the attending's ability to keep the patient safe to focus on learning; 3) Instructional Approach: Use of specific and directional language, readiness to teach components of a case, and willingness to figure out what is preventing the resident from following directions; 4) Feedback Content and Delivery: Whether feedback is actionable, constructive (explains why/how), specific vs. vague, and delivered timely (IO for technical, PO debrief for overall). The distinction between "good" and "bad" yelling; 5) Discernment of Resident Needs: Attending's ability to manage expectations consistently, individualize instruction based on resident skill and stress level, and grant appropriate autonomy while explaining takeovers; 6) Variety of Teachers: Exposure to diverse teaching styles and methods is seen as beneficial for becoming a well-rounded surgeon; 7) Relationship/Environment: A respectful, collegial relationship and a calm, safe environment (vs. hostile/volatile) are crucial for learning and receiving feedback effectively. The team environment is also important. | Resident perception was measured using a qualitative research approach based on grounded theory. Data was collected through five in-person semi-structured focus groups with general surgery residents. The focus group discussions explored residents' perspectives on effective IO faculty teaching qualities. The audio recordings were transcribed and analyzed thematically using an inductive coding process to identify recurring themes and subthemes representing the residents' shared perceptions. |
| 01/2024 | Chief residents perceive the OC program and its feedback as effective in improving their self-regulated learning and promoting confidence and opportunities for autonomy. They particularly value neutral authentic feedback, third-party real-time observation (from the coach), and actionable feedback that provides specific, poignant guidance. Residents tend to underestimate their own surgical skills compared to attendings, but their self-graded PE scores align with coaches and attendings. They perceive that attending surgeons tend to overestimate the autonomy they permit. Despite differences in perception of skill/autonomy between residents, coaches, and attendings, they reach consensus on resident PE upon graduation. Qualitative feedback suggests residents find OC feedback different and more helpful than normal evaluations. | Residents perceive the OC program and its feedback positively in several ways: 1) Effectiveness: Consistently described as effective in promoting confidence and opportunities for autonomy; 2) Valued Elements: Specifically value neutral authentic feedback, third-party real-time observation, and actionable feedback; 3) Specificity and Usefulness: Appreciated specific, poignant feedback that changed the way they operated. Third-party observation was seen as helpful for pointing out details attendings might miss; 4) Alignment with Peers/Attendings: Residents' self-graded PE scores align with coaches and attendings upon graduation. Some residents also noted their assessment differences with the coach converged over time; 5) Promoting Self-Improvement: Some residents intentionally rated themselves lower than attendings/coaches as a way to motivate self-improvement, without undermining their confidence. | The study identifies a perceived self-efficacy deficit where chief residents on average underestimate their surgical skills (PSS and GS) compared to attending surgeons. Attendings are also perceived as tending to overestimate the operative autonomy they permit to residents. While not directly negative about the OC feedback *process* itself, these discrepancies in perception highlight potential areas for misinterpretation or misalignment between resident and faculty views that OC might help address. Some potential explanations from residents/faculty interviews touch on potential negatives, such as attendings grading differently, or grades potentially being influenced by personal relationships rather than hard qualifications, or inconsistent case selection by residents. The limitation section notes that SEPAs assessments can be subject to bias (gender, racial). | Resident perception of the OC program and its feedback is influenced by: 1) Source of Observation: Real-time, third-party observation by a dedicated coach is highly valued and provides insights attendings might miss; 2) Feedback Characteristics: Feedback that is neutral, authentic, specific, and actionable is perceived as most effective and valuable; 3) SEPAs Evaluation: The structured use of SEPAs provides a framework for evaluation and feedback. Reviewing aggregated reports might influence perceptions of alignment; 4) Relationship with Evaluators: The perceived relationship with coaches and attendings can influence how feedback and evaluations are interpreted. The consensus on PE upon graduation suggests a degree of shared understanding is achieved. 5) Self-Reflection Tendency: Residents' tendency to be self-critical (underestimating skills) influences their self-perception compared to others; 6) Attending Behavior: Attendings' grading variations, perception of autonomy granted, and potentially their comfort/experience with giving feedback can influence the resident's overall experience and perception of the evaluation process; 7) Training Level: The study focuses on chief residents, and their perspective might differ from junior residents regarding skill progression and entrustment. | Resident perception was measured through a mixed-methods approach: 1) Qualitative Interviews: Semi-structured interviews with graduated chief residents specifically explored their experiences with and perspectives on the OC program, the SEPAs instruments, and which OC elements they found most effective; 2) Qualitative Text Analysis: Written comments provided by chief residents within the SEPAs evaluations were analyzed for recurring terms and themes, giving insight into their reflections and perceptions expressed in writing; 3) Quantitative Comparison of Ratings: The study compared average SEPAs ratings (including PE, PSS, GS, SSG) provided by chief residents (self-assessment) against those provided by faculty coaches and attending surgeons. Discrepancies in these ratings represent differences in perception. |

Table 5. Impact of feedback on residents' education

| Study ID | Impact on learning progress | Impact on quality of patient care (if mentioned) | Impact on learner engagement (if mentioned) | Impact on skill development | Impact on self-confidence | Impact on reflection/internal feedback |
| --- | --- | --- | --- | --- | --- | --- |
| 01/2017 | The study demonstrated an overall improvement in residents' attitudes and practice, with a statistically significant increase in mean scores after the feedback intervention. This was evidenced by the mean difference of 0.43 ± 0.06 in overall scores between the two surveys. Furthermore, feedback led to specific behavioral changes, such as reduced smoking and increased time spent on studies by some residents. | Not mentioned | Not mentioned | MSF is recognized for assessing non-technical competencies such as communication skills, interpersonal skills, collegiality, humanism, and professionalism. The questionnaire used in the study specifically addressed communication and interpersonal skills, resource utilization, reliability, professionalism, and response to adverse situations. The improvement in overall scores suggests a positive impact on the development of these skills and attitudes. | Not mentioned | Not mentioned |
| 02/2017 | Not mentioned | Not mentioned | Not mentioned | Not mentioned | Not mentioned | Not mentioned |
| 01/2018 | Not mentioned | Not mentioned | Not mentioned | Not mentioned | Not mentioned | Not mentioned |
| 02/2018 | Not mentioned | Not mentioned | Not mentioned | Not mentioned | Not mentioned | Not mentioned |
| 01/2019 | Not mentioned | Not mentioned | Not mentioned | Not mentioned | Not mentioned | Not mentioned |
| 01/2020 | Not mentioned | Not mentioned | Not mentioned | Not mentioned | Not mentioned | Not mentioned |
| 02/2020 | Not mentioned | Not mentioned | Not mentioned | Not mentioned | Not mentioned | Not mentioned |
| 01/2022 | Not mentioned | Not mentioned | Not mentioned | Not mentioned | Not mentioned | Not mentioned |
| 02/2022 | Not mentioned | Not mentioned | Not mentioned | Not mentioned | Not mentioned | Not mentioned |
| 01/2023 | Not mentioned | Not mentioned | Not mentioned | Not mentioned | Not mentioned | Not mentioned |
| 02/2023 | Not mentioned | Not mentioned | Not mentioned | Not mentioned | Not mentioned | Not mentioned |
| 01/2024 | The OC program and its associated feedback are perceived by residents as positively impacting their learning progress. It's described as effective in improving self-regulated learning. Feedback, particularly when specific and actionable, helps residents gain awareness of their strengths/weaknesses, prepare learning goals, and employ strategies to achieve them. The progression of resident skill (GS), autonomy (SSG), and entrustment (PE) throughout the year, assessed within the OC program, indicates positive learning progression. |  |  | The OC program and its feedback are directly aimed at improving residents' operative skills (PSS, GS) and autonomy (SSG). The study demonstrates significant improvement in GS and SSG throughout the chief year within the OC program. Residents valued feedback as changing the way they operated, directly linking it to skill improvement. The program focuses on procedure-specific SEPAs, reinforcing the link to specific skill development. | The impact on self-confidence is mentioned. Graduated chief residents consistently described the OC program as effective in promoting their confidence. One resident specifically stated that even though they had less autonomy in fellowship, the coaching program "gave me confidence." Interestingly, residents' tendency to underestimate their skills did not appear to undermine their self-confidence in the study. | The OC program and its feedback facilitate resident reflection and self-regulated learning. Residents' self-assessment via SEPAs evaluations, coupled with coach and attending evaluations, provides data for reflection. Chiefs described OC as effective in improving their self-regulated learning. Feedback helps residents gain awareness of their strengths and weaknesses, which is crucial for internal reflection and setting personal learning goals. The text analysis of residents' written reflections in SEPAs also provides evidence of their internal processing. |

Table 6. Knowledge gaps and implications

| Study ID | Knowledge gaps identified by the study authors | Implications for practice | Suggestions for future research |
| --- | --- | --- | --- |
| 01/2017 | The authors identified that, although there is awareness about MSF among Pakistani medical educationists, very little research has been done in this area. Specifically, the workplace-based assessment (WBA) tool of MSF had not yet been tested in the Pakistani context. | The study suggests that MSF, when delivered accurately, timely, with confidentiality, and facilitative feedback sessions, can lead to an improvement in residents' practice. This implies that MSF is a valuable tool for postgraduate medical education. Maintaining the confidentiality of raters is crucial for the success of the process, and implementing MSF may require management commitment and specific training to handle workload and resistance to change. | The authors suggest that further studies are needed to determine the role of MSF specifically in the context of Pakistani doctors. The current study serves as a baseline for future research in this field. Additionally, one of the participating residents proposed that similar multisource feedback sessions should also be conducted for faculty members, which could be a direction for future investigations. |
| 02/2017 | The authors identified several gaps in literature and knowledge regarding post-operative feedback in surgical education: 1) Little is known about how to consistently deliver PO feedback; 2) Further study is needed to validate resident and attending perceptions of IO and PO feedback; 3) No work has been done to gauge the success of interventions designed to improve feedback compared to formal PO feedback; 4) The study design did not assess the perceived quality of current feedback versus the ideal quality of feedback; 5) The study reflects the perceptions of only one department in one academic institution, limiting generalizability and highlighting a gap in understanding potential differences in barriers based on program type; 6) The study did not address specific methods for delivering PO feedback or specific content that should be covered. | The findings carry several implications for current surgical education practice: 1) PO feedback is currently provided much less frequently than perceived as ideal by both residents and staff; 2) PO workflow needs to be modified to effectively incorporate this crucial teaching moment; 3) There needs to be a cultural shift in surgical practice to recognize PO feedback as an integral part of patient care, not secondary to it; 4) Greater emphasis should be placed on the importance of non-technical skills and their link to patient outcomes, potentially through education for both residents and staff; 5) Training staff on how to effectively assess and provide feedback for non-technical skills is suggested; 6) Efforts should be made to address the identified barriers (time, competing responsibilities, privacy/environment, interest) to facilitate more frequent and effective feedback. | The authors propose the following avenues for future research based on their findings and identified gaps: 1) Future work should study IO and PO feedback to validate resident and attending perceptions. This validation is necessary to develop effective interventions to improve and facilitate the feedback process; 2) Studies should evaluate varying departments across multiple teaching institutions, including academic and community programs. This would enhance the generalizability of findings and provide insight into how barriers may differ across different training environments; 3) Future research should include qualitative methods like interviews or focus groups to gain deeper understanding of ideal feedback delivery methods and the specific content that should be included in feedback; 4)Future studies should assess the perceived quality of feedback, both current and ideal, which was a limitation of the present study. |
| 01/2018 | The authors identified several knowledge gaps in the literature and surgical education landscape: 1) Limited evidence on the best way to deliver feedback to surgical trainees; 2) Lack of consensus between faculty and trainees regarding trainee learning needs; 3) Lack of consensus between faculty and trainees regarding the components of high-quality feedback; 4) Lack of consensus between faculty and trainees regarding the amount of feedback given; 5) Lack of consensus between faculty and trainees regarding how feedback is interpreted; 6)The study itself only examined the trainees' perspective, not the preceptor's, which is a limitation acknowledged by the authors. | The study findings have practical implications for surgical educators and training programs: 1) Improve Operative Training: The results can inform the design and implementation of interventions aimed at improving operative training methods; 2) Incorporate Trainee Perspective: It is crucial to incorporate the trainee perspective when trying to improve the quality of feedback; 3) Optimize Performance Rating Tools: If using performance rating tools, programs should carefully select tools that encourage face-to-face feedback, employ accurate and meaningful behavioral anchors, and ensure evaluators and trainees are effectively trained on their use and interpretation. | Based on their limitations and findings, the authors implicitly and explicitly suggest directions for future research: 1) Preceptor Perspective: Future studies should examine feedback from the perspective of the preceptor (faculty/attending surgeons) to get a more complete picture; 2) Larger/More Targeted Samples: Larger sample sizes or more directed interviews might be needed to fully capture the diversity of views on feedback attitudes and needs, and to explore additional relevant topics not fully covered; 3) Subgroup Comparisons: Future research could compare views and experiences across different subgroups of trainees (e.g., by surgical specialty, specific PGY levels) at a more detailed level, which was limited in this study; 4) Intervention Studies: Research is needed on interventions to enhance feedback efficiency and efficacy; 5) Achieving Consensus: Studies exploring strategies to bridge the consensus gap between faculty and trainees regarding learning needs, high-quality feedback components, amount of feedback, and interpretation. |
| 02/2018 | The authors implicitly identify the following knowledge gaps or areas needing further exploration: 1) The need to understand the factors influencing confidence from the *residents' perspective*, which was the primary aim of this qualitative study, suggesting previous research might have been more researcher-driven; 2) The lack of understanding of factors contributing to the perceived "confidence crisis" among general surgery residents; 3) A limitation of their own study is that they did not examine feedback (or confidence development factors) from the *preceptor's perspective*; 4) The need to explore if gender influences on confidence might be more noticeable with more direct questioning, implying this is a gap in the current understanding or methods. | The study findings have direct implications for educators in surgical training: 1) Improving Learning Experiences: Understanding the internal and external factors affecting resident confidence can help educators improve learning experiences; 2) Accelerating Progress: By addressing factors that build confidence, educators can accelerate residents' progress towards becoming confident, independent surgeons; 3) Focus on Constructive Feedback: Knowing that constructive feedback has a large impact on resident confidence, programs and staff surgeons should implement changes to improve the quality and frequency of feedback, ensuring it explains *why* and *how* to improve; 4) Cultivating Supportive Environments: Recognizing the importance of supportive environments and positive rapport, educators should foster such relationships. | The authors suggest several areas for future research: 1) Replication with Larger Sample: Repeating the study with more residents from general surgery and/or other surgical specialties and institutions to address the limitation of a small sample from a single institution; 2) Including PGY 1 and PGY 5: Exploring the confidence experiences of PGY 1 and PGY 5 residents, who were excluded from this study; 3) Exploring Preceptor Perspective: Future qualitative research should investigate the factors affecting confidence from the viewpoint of the preceptor/faculty; 4) More Directed Questioning on Gender: Future studies could use more direct questioning to better understand the potential impacts of gender on confidence; 5) Quantitative Validation: While a qualitative study, the findings could potentially inform future quantitative studies on confidence factors. |
| 01/2019 | The authors identify several areas requiring further understanding: 1) Previous survey-based studies lacked the ability to describe feedback types accurately or identify nonverbal forms, a gap this study aimed to address using video analysis; 2) Further studies are needed to fully characterize the finding that residents showed no significant difference in overall satisfaction despite perceiving lower frequency, specificity, and immediacy of feedback; 3) Additional studies are necessary to definitively delineate *why* residents are less likely to identify certain IO interactions (especially nonverbal and unlabeled ones) as feedback, beyond the hypothesis that it might be perceived as teaching not aligned with their goals; 4) More studies with larger sample sizes are needed for alternative interval analysis in video review and subgroup analysis (which their study was limited in performing); 5) Further studies are necessary to determine if the PO debrief, particularly within frameworks like the BID (briefing, IO teaching, debriefing) model, truly results in improved alignment of expectations regarding feedback between residents and faculty. | The findings have practical implications for improving surgical education: 1) Labeling Feedback: Explicitly labeling IO interactions as "feedback" (both verbal and nonverbal) may improve resident recognition and reception of feedback; 2) PO Review: Incorporating immediate PO review of performance may be more effective for feedback delivery and reception, as residents appear more receptive at this time; 3) Using Structured Models: Implementing structured teaching models like the BID model can help integrate feedback more intentionally and address some of the identified disparities in perception and timing; 4) Video Review as a Tool: Video review is a valuable methodology for studying feedback perceptions and can be applied in other medical education contexts. | The authors suggest the following avenues for future investigation: 1) Further studies to understand why residents are satisfied despite perceiving less feedback frequency/specificity/immediacy; 2) Studies to delineate the specific reasons residents fail to identify certain interactions, like nonverbal cues or unlabeled comments, as feedback; 3) Research using video review with larger sample sizes to enable subgroup analysis (e.g., by PGY level, specialty) and different interval analyses; 4) Studies evaluating the impact of PO debriefs, particularly using video review to see how perceptions of the debrief differ between faculty and residents; 5) Research applying the video-based perception analysis methodology to other clinical learning environments. |
| 01/2020 | The authors identify several knowledge gaps and areas needing further study: 1) The reasons why differences exist between the frequency of feedback perceived at university and community programs need to be elucidated; 2) It is necessary to ascertain whether resident perceptions of feedback (e.g., amount received, recognition as feedback) match the reality from the faculty perspective; 3) Their study did not assess faculty's comfort and experience with providing feedback, indicating this as a gap in understanding; 4) Understanding attending surgeons' preparedness regarding providing feedback is needed; 5) The optimal methods of teaching and providing feedback in surgical education need to be ascertained; 6) Their study's limitations (small sample size from a single urban system, single specialty) suggest a gap in broader understanding across different contexts and specialties; 7) The terms used for the survey ("feedback," "constructive") were not explicitly defined for participants, which is noted as a limitation that could introduce variability; 8) The specific cultural or sub rosa factors contributing to the observed differences between community and university settings need further exploration. | The findings have several implications for improving the practice of surgical education: 1) Surgical training programs should prioritize improving feedback delivery, particularly in the PrO and PO phases where it is perceived as most lacking; 2) Instituting structured formats and designated times for feedback sessions (pre-briefing and post-debriefing) is strongly supported by resident preferences and should be considered; 3) Faculty development programs focusing on effective feedback delivery, including explicit labeling of feedback and creating a safe environment, are recommended; 4) Leveraging the perceived value of feedback from senior residents could be explored within the educational structure; 5) Programs should strive to foster a supportive learning environment where residents feel safe to ask questions; 6) Identifying best practices from settings where feedback is perceived as more frequent (like the community hospitals in this study) could inform improvements elsewhere; 7) Promoting a broader "feedback culture" is essential; 8) Training residents to actively seek feedback in a supportive environment is also suggested. | Based on the identified gaps and limitations, the authors suggest the following areas for future research: 1) Studies surveying faculty members to understand their perceptions and preparedness regarding providing feedback; 2) More expansive studies with larger sample sizes and inclusion of multiple institutions (beyond a single urban system) and surgical specialties (beyond general surgery) to gain broader insights; 3) Research specifically aimed at elucidating the reasons for the observed differences in perceived feedback frequency between university and community hospital settings; 4) Studies designed to ascertain the congruence between resident and faculty perceptions of the amount and recognition of feedback; 5) Research focused on identifying the most effective methods for teaching and providing feedback in surgical education; 6) Studies evaluating specific interventions or structured models (like BID) aimed at improving the frequency, quality, and reception of feedback; 7) Research investigating the factors that influence faculty comfort and experience in providing feedback; 8) Studies that use more explicitly defined terms for feedback to participants; 9) Research exploring strategies to train residents in feedback-seeking behavior and the characteristics of supportive learning environments. |
| 02/2020 | The authors identify the following knowledge gaps: 1) It is unclear how feedback on leadership performance is currently delivered during surgical residency; 2) It is unknown whether formal ACGME assessments result in adequate feedback for leadership development; 3) While many studies exist on feedback for clinical/technical skills, specific examination of feedback practices around *leadership* performance is lacking; 4) Their study was exploratory qualitative research conducted in a single program, limiting generalizability; 5) Their study focused only on the residents' perspective, lacking the perspectives of faculty or other team members; 6) Feedback emerged as a topic but was not the primary aim of the initial interviews, suggesting a need for further exploration specifically on how residents receive leadership feedback or handle hurtful comments. | The study findings have several implications for improving leadership feedback in surgical residency: 1) Formal assessment of leadership skills should be implemented as part of resident evaluation, with feedback provided from multiple sources (e.g., 360-degree tools); 2) Programs need to address and overcome structural (opportunity, hierarchy) and personal/cultural barriers (reluctance to criticize, taking feedback personally) to giving and receiving honest, constructive leadership feedback; 3) Strategies to promote a positive and open feedback environment, such as building trusting relationships, focusing on future growth, and making feedback systematic, are important; 4) Using a coaching model, pairing residents with trusted mentors to review leadership feedback and develop action plans, could be effective; 5) Building a formal leadership feedback mechanism into the residency culture can set expectations, facilitate openness, and reduce defensiveness; 6) Improving the culture around leadership-specific feedback may also benefit general feedback practices. | The authors suggest the following directions for future research: 1) Studies examining feedback from the perspective of faculty members, nurses, advanced practice providers, and other team members; 2) Research specifically focused on leadership-specific feedback, delving deeper into how residents receive feedback and how they handle critical or hurtful comments; 3) Studies conducted in multiple residency programs and institutions to improve the generalizability of findings; 4) Investigations into the specific interventions based on resident recommendations (e.g., implementing formal feedback mechanisms, mentorship programs, training on giving/receiving leadership feedback) and evaluating their effectiveness. |
| 01/2022 | The authors explicitly state limitations that point to knowledge gaps: 1) Only written feedback was evaluated, not verbal feedback occurring during the case; 2) The study could not ask follow-up questions to faculty (to understand their intent) or residents (to understand *why* they rated feedback as they did); 3) Given the multi-center nature and spectrum of experience, there was no common frame of reference for feedback understanding prior to implementing SAP; 4) While the study provides insight into *what* feedback types are valued, it doesn't fully explain *why* residents find some types more valuable than others, suggesting a need for additional in-depth analysis; 5) The study focused on the resident perspective and did not include the faculty/preceptor's perspective on feedback delivery or perception. | The findings offer concrete implications for faculty providing written operative feedback: 1) Prioritize providing written comments that are specific to the resident's performance, include key learning points, or indicate progress towards independence (themes: specific general observation, key points, independent practice); 2) Avoid leaving the written comment section blank, but more importantly, avoid non-specific comments; 3) Absolutely avoid comments indicating a lack of recall of the resident's performance ("I don't remember you..."); 4) Consider using a theory-based framework like ZPD (or tools based on it like SAP) to structure observations and feedback; 5) Do not focus *solely* on "next steps"; contextualize future goals with current performance or key learning points; 6) Aim for timely completion of assessments to maximize the perceived quality and value of the feedback; 7) Faculty training in providing specific, theory-informed feedback is recommended. | The authors suggest the following areas for future investigation: 1) Conduct additional in-depth analysis, likely qualitative, to explore *why* residents perceive certain types of feedback (e.g., specific themes vs. non-specific or blank) as more valuable; 2) Future studies should include the perspective of faculty/preceptors regarding feedback practices and perceptions; 3) Research incorporating analysis of verbal feedback that occurs during the operative case, in addition to written feedback; 4) Studies to better understand the impact of the relationship between faculty and residents on feedback perception; 5) Investigation into effective faculty development programs to improve feedback quality. |
| 02/2022 | The authors identify several limitations that suggest knowledge gaps: 1) The study was conducted at a single tertiary medical center, potentially limiting the generalizability of findings to other institutions; 2) The sampling approach limited the number of faculty categorized, potentially not reflecting the full range of experiences; 3) The reliance on resident evaluations introduces subjectivity and potential reporting bias (evaluations not consistently reported by all trainees) and recall bias (evaluations collected at various times); 4) OpTrust® measures were collected only during the operation, while resident comments covered the entire service rotation, creating a potential discrepancy in the time frame of observed behaviors vs. reported perceptions; 5) Due to the small sample size for categorical analysis, they were unable to perform detailed subgroup analysis within the "promoting" and "limiting" categories (e.g., between good and excellent promoters, or moderate and low limiters) to understand more nuanced behaviors; 6) Specific faculty behaviors associated with resident advancement were not well identified in the literature prior to this study. | The findings have significant implications for surgical educators and training programs: 1) Efforts to improve resident operative performance and autonomy should focus not only on technical teaching but also on faculty's personal traits and the learning environment they create; 2) Faculty development programs should incorporate training on effective teaching techniques, including structured PrO goal setting, PO debriefing, and providing constructive, specific feedback; 3) Training should emphasize creating a supportive, respectful, and collaborative operating room environment; 4) Faculty should be mindful of how their personal demeanor and communication style are perceived by residents, as these heavily influence the learning experience; 5) Mentorship and surgical coaching programs are recommended as ways to support faculty in developing their educational skills and promoting resident growth; 6) Using tools like OpTrust® and reviewing the data from teaching evaluations can help faculty identify areas for personal and teaching improvement. | The authors suggest the following areas for future research: 1) Studies with larger sample sizes from multiple institutions to improve the generalizability of the findings; 2) Further investigation to explore the nuances of faculty behaviors by stratifying promoting and limiting faculty into sub-groups (e.g., highly promoting vs. moderately promoting); 3) Research examining the perspectives of faculty and other operating room team members (nurses, scrub techs, etc.) on these interactions, to complement the resident perspective; 4) Studies evaluating the impact of specific faculty development interventions on resident perceptions and outcomes; 5) Further research on continuous professional development programs for surgeons as educators, focusing on entrustment and promoting behaviors. |
| 01/2023 | The authors identify several knowledge gaps and areas requiring further study: 1) Limited data exists evaluating the experience of surgical subspecialty residents on general surgery rotations; 2) No studies to their knowledge had examined these off-service rotations for surgical subspecialty residents *within a single institution* prior to their study; 3) It is "not clear why" surgical subspecialty residents perform fewer cases than their general surgery counterparts; 4) The exact reasons behind the discrepancy in case numbers are "unknown." Potential explanations need further exploration (e.g., less opportunities to scrub, more time for floor work, less initiative); 5) Additional work is needed to further explore the question of *why* subspecialty residents log fewer cases than categorical general surgery residents, beyond faculty perceptions; 6) The limitations of their study (single center, small total case log sample size, low survey response rates especially for female and junior residents, reliance on variable case log data accuracy) highlight areas needing more robust research; 7) Despite off-service rotations being examined in other specialties, "further information is still needed regarding off-service rotations for surgical subspecialty residents." | The study findings have implications for improving the off-service rotation experience for surgical subspecialty residents: 1) Core surgical rotations are confirmed as an important component of subspecialty training and help prepare residents for their careers; 2) Efforts should focus on enhancing learning opportunities for subspecialty residents, particularly given the lower operative volume compared to general surgery residents; 3) Focusing on aspects other than operative volume, such as working as a team member, valuing resident opinions, and providing faculty feedback and mentorship, is important as residents find value in these areas; 4) Institutions should consider modifications to rotations to improve the subspecialty resident experience; 5) Strategies to increase non-operative productivity measures could be explored; 6) Consider providing a more defined curriculum and stated objectives for each rotation, focusing on those with significant case number differences; 7) Program directors and faculty should be aware that feedback is perceived as beneficial for learning and associated with increased satisfaction on off-service rotations. | The authors suggest several areas for future investigation: 1) Future work should focus on further investigating the discrepancy in case numbers between general surgery and subspecialty residents on core rotations; 2) Research should aim at enhancing learning opportunities for subspecialty residents; 3) Studies could explore why some subspecialty residents (especially urology residents in their data) may not accurately log cases during these rotations; 4) Additional research is needed to explore the potential reasons behind the case number discrepancy, potentially including faculty and resident perspectives beyond what was captured in their survey; 5) Studies could evaluate whether specific interventions (e.g., defined curricula, objective setting, tracking non-operative productivity) improve the off-service rotation experience and case volumes for subspecialty residents; 6) Investigate additional ways to evaluate whether subspecialty residents are effectively learning core surgical skills and knowledge on these rotations (e.g., analyzing ABSITE (American Board of Surgery In-Training Examination) scores, evaluating competency via skills labs or oral exams before and after rotations); 7) Multi-center studies are needed to assess the generalizability of their findings regarding case volumes and perceptions; 8) Studies with larger and more representative survey sample sizes (especially for female and junior residents) are needed. |
| 02/2023 | The authors identify several knowledge gaps and areas needing further study: 1) The perspective of the learner (resident) on IO teaching has been less explored compared to the expert educator viewpoint; 2) Prior sources (frameworks, expert input) lack the trainee perspective, potentially not addressing resident needs; 3) Faculty's ability to accurately identify learner needs is reportedly limited, despite reporting its importance. There is a significant difference between faculty and resident assessments of teaching and educational priorities; 4) Their study was conducted at a single academic center, limiting generalizability; 5) Their study only included general surgery residents, lacking perspectives from other surgical disciplines; 6) Their study only included residents, lacking the perspective of faculty members on their expectations and barriers regarding teaching; 7) The notion of valuing a diversity of teaching styles is novel and not widely reported in the literature, suggesting this is a knowledge gap. | The findings offer practical implications for surgical educators and training programs: 1) Emphasize the importance of the teacher's character, including demonstrating genuine care for the learner, showing respect, and maintaining self-control, as these are foundational to effective teaching; 2) Encourage the use of tangible strategies for feedback, such as using clear directional words and giving actionable feedback that explains *why* and *how* to improve; 3) Implement timely debriefing sessions, especially after challenging cases, to consolidate learning and set goals; 4) Recognize the importance of individualizing instruction based on resident needs and skill levels, including knowing when to allow productive struggle and when to take over, and explaining the rationale for actions; 5) Training programs should celebrate and encourage diversity of personas and teaching styles among faculty rather than promoting a single idealized style; 6) Faculty development should focus on these tangible strategies and the ability to discern and respond to individual learner needs. | Based on their findings and limitations, the authors suggest the following areas for future investigation: 1) Compare the differences in perceptions of effective IO teaching between junior and senior surgical residents; 2) Interview faculty members to understand their expectations regarding teaching in the OR and perceived barriers to implementing best teaching practices; 3) Conduct studies in multiple institutions and potentially other surgical disciplines to explore perceptions more broadly and see if additional themes emerge; 4) Investigate coaching strategies that can help faculty develop their personal teaching styles effectively; 5) Research on interventions aimed at bridging the gap between faculty self-assessment and resident assessments of teaching. |
| 01/2024 | The authors identify several knowledge gaps and limitations that indicate areas needing further study: 1) Little was known about how surgery residents' operative skills and PE progress within an OC program throughout the chief year prior to their study; 2) Little was known about what specific OC elements are most effective or helpful from participants' perspectives prior to their study; 3) Their study was conducted at a single institution, limiting generalizability; 4) The quantitative SEPAs evaluations are subjective rater-based assessments, potentially affected by cognitive bias; 5) The qualitative sample size for interviews is relatively small; 6) Social desirability bias may influence interviewees' impressions and comments; 7) Their study is not designed to demonstrate whether OC *itself* improves practice readiness over other teaching methods due to the lack of a pre-OC program benchmark or a non-OC comparison group using similar assessment methods; 8) Gender and racial differences in trainee, coach, or attending SEPAs assessments were *not* explored in this study, despite emerging literature indicating bias in entrustability assessments. | The findings have significant implications for surgical training programs: 1) Implementing procedure-specific SEPAs, especially within an OC program framework, can be a valuable approach for assessing and promoting resident procedural skills, autonomy, and PE; 2) Autonomy (ability to complete case-specific steps without guidance) is key to developing entrustment and competence, suggesting interventions should focus on increasing supervised autonomy; 3) OC, and the use of SEPAs, can enhance both resident learning and faculty teaching in the operating room; 4) Faculty can use aggregated SEPAs evaluations and written comments to adjust their teaching plans to the needs of a particular case and resident; 5) Training programs may consider implementing or enhancing structured coaching programs that include real-time observation by a third party and focus on providing neutral authentic and actionable feedback based on objective performance measures like SEPAs; 6) Addressing and understanding potential self-efficacy deficits (residents underestimating skills, attendings overestimating autonomy granted) is important. | The authors suggest several areas for future investigation: 1) Studies with larger qualitative sample sizes are needed to further validate the findings regarding participant perceptions; 2) Future studies should specifically investigate gender and racial patterns in SEPAs assessments and OC data to identify and address potential biases; 3) Research is needed that compares outcomes (e.g., practice readiness) between residents participating in OC programs versus those in traditional training methods, ideally with pre-intervention benchmarks; 4) Studies could explore the specific administrative, logistical, and financial hurdles of implementing OC programs in different settings; 5) Further investigation into cognitive bias in subjective rater-based assessments like SEPAs; 6) Research exploring why residents underestimate their skills and attendings overestimate autonomy granted, and the impact of these self-efficacy deficits. |

List of abbreviations:

ABSITE: American Board of Surgery In-Training Examination

ACGME: Accreditation Council for Graduate Medical Education

ADT: Academic Development Time

ALG: Achieved Learning Goal

BID: Briefing, Intraoperative Teaching, Debriefing

COREQ: Consolidated Criteria for Reporting Qualitative Research

FCPS: Fellowship of the College of Physicians and Surgeons

GS: General Skill

IO: Intraoperative

IT: Information Technology

MSF: Multi-Source Feedback

NCC: National Capital Consortium

OC: Operative Coaching

OR: Operation Room

PAT: Peer Assessment Tool

PE: Prospective Entrustment

PGRs: Postgraduate Trainees

PGY: Postgraduate Year

PO: Postoperative

PrO: Preoperative

PSS: Procedural-Specific Skill

REDCap®: Research Electronic Data Capture

SAP: Surgical Autonomy Program

SEPAs: Surgical Entrustable Professional Activities

SPSS®: Statistical Package for the Social Sciences

SSG: Step-Specific Guidance

TAGS: Teach and demonstrate, Advise and scaffold, Guide and monitor, Solo and observe

USA: United States of America

ZPD: Zone of Proximal Development

WBA: Workplace-Based Assessment
